# Ototoxicity-induced c-Fos activation underlies the regenerative capacity of the vestibular sensory epithelia

**DOI:** 10.1186/s12964-025-02446-y

**Published:** 2025-10-08

**Authors:** Yunzhong Zhang, Dan You, Chenhao Che, Xinyuan Wang, Huawei Li, Yi-Quan Tang, Shan Sun

**Affiliations:** 1 ENT Institute and Otorhinolaryngology, Department of Affiliated Eye and ENT Hospital, Key Laboratory of Hearing Medicine of NHFPC, Shanghai Key Laboratory of Gene Editing and Cell Therapy for Rare Diseases, State Key Laboratory of Medical Neurobiology, Fudan University, Shanghai, 200031 China; 2 Institutes of Brain Science, Fudan University, Shanghai, 200032 China

**Keywords:** c-Fos, Hair cell regeneration, AP-1 transcription factor, Notch signaling, Wnt signaling, Atoh1, Vestibular function, Inner ear

## Abstract

The mammalian inner ear exhibits a striking dichotomy in regenerative capacity: while vestibular hair cells retain limited ability to regenerate, cochlear hair cells permanently lose this potential after birth. Exploiting this disparity, we employed single-cell comparative transcriptomics combined with lineage tracing to identify c-Fos as a critical early responder selectively activated in the vestibular epithelium following ototoxic injury. Transcriptomic and chromatin profiling revealed that c-Fos initiates regenerative reprogramming by transcriptionally priming *Atoh1*, the master regulator of hair cell fate, while synergizing with the Wnt and Notch pathways to balance progenitor proliferation and trans-differentiation. Functional validation showed that c-Fos overexpression significantly promoted vestibular hair cell regeneration and restored balance function in adult mice. Our findings establish c-Fos as a molecular switch bridging acute injury responses to tissue repair and highlight its potential as a therapeutic target for vestibular rehabilitation.

**Supplementary Information:**

The online version contains supplementary material available at 10.1186/s12964-025-02446-y.

## Introduction

Hearing and balance disorders caused by the irreversible loss of sensory hair cells in the cochlea and vestibular system represent a significant global health burden, affecting over 1.5 billion people worldwide. In mammals, including humans, the regenerative capacity of inner ear hair cells exhibits a striking dichotomy: vestibular hair cells retain partial regenerative potential, while cochlear hair cells lose this capacity postnatally. This disparity has profound clinical implications, as cochlear hair cell loss underlies most cases of sensorineural hearing loss, whereas vestibular dysfunction contributes to severe symptoms such as vertigo and imbalance. Elucidating the molecular and cellular mechanisms underlying this divergence is critical for developing regenerative therapies.

Early studies have shown that non-mammalian vertebrates can regenerate hair cells via supporting cells proliferation and trans-differentiation [1]. For instance, birds are able to replace lost hair cells by reactivating cell‐cycle pathways [2–4]. In contrast, mammalian cochlear supporting cells exit the cell cycle during early development and remain terminally differentiated. In the mammalian cochlea, persistent expression of cell cycle inhibitors such as p27^Kip1^ and retinoblastoma protein locks supporting cells in a quiescent state, thereby preventing them from re‐entering the cell cycle after damage [5–7]. However, vestibular supporting cells in mammals appear to be less stringently regulated. Although they also express cell cycle inhibitors, they retain a limited ability to proliferate and differentiate into hair cells after injury [8, 9]. These findings suggest intrinsic differences in the regulatory mechanisms governing the two sensory epithelia.

At the molecular level, the transcription factor *Atoh1* is essential for hair cell differentiation [10, 11]. In the cochlea, *Atoh1* expression is tightly suppressed in mature supporting cells, likely contributing to their failure to trans-differentiate into hair cells. In contrast, the vestibular epithelium provides a more permissive environment for *Atoh1* reactivation, enabling partial hair cell regeneration following damage [12]. Additionally, differences in the activity of signaling pathways—such as Notch and Wnt—further contribute to this regenerative disparity. In the cochlea, Notch signaling reinforces the supporting cell phenotype, while in the vestibule Wnt pathway activation may promote regeneration by facilitating both cell proliferation and trans-differentiation [13]. Despite these insights, the mechanisms by which ototoxic damage initiates vestibular but not cochlear regeneration remain poorly understood.

In this study, we performed single-cell comparative transcriptomics on acutely damaged cochlear and utricular tissues to identify gene signatures associated with vestibular repair. Our analysis revealed that c-Fos, a canonical immediate-early gene [14], is pivotal for vestibular tissue repair and cell survival [15]. Following ototoxic injury, vestibule-specific c-Fos activates *Atoh1* and synergizes with Wnt/Notch signaling to balance progenitor cell proliferation and trans-differentiation [16, 17]. Overexpression of c-Fos via intratympanic delivery in adult mice leads to robust utricular hair cell regeneration and functional recovery. These findings establish c-Fos as a molecular switch linking acute injury responses to regenerative reprogramming in the vestibular epithelia, thus underscoring distinct repair mechanisms in vestibular versus cochlear systems and suggesting new therapeutic targets for vestibular disorders.

## Results

### Ototoxic injury induces *Fos* expression in the utricle but not the cochlea

**Fig. 1:**
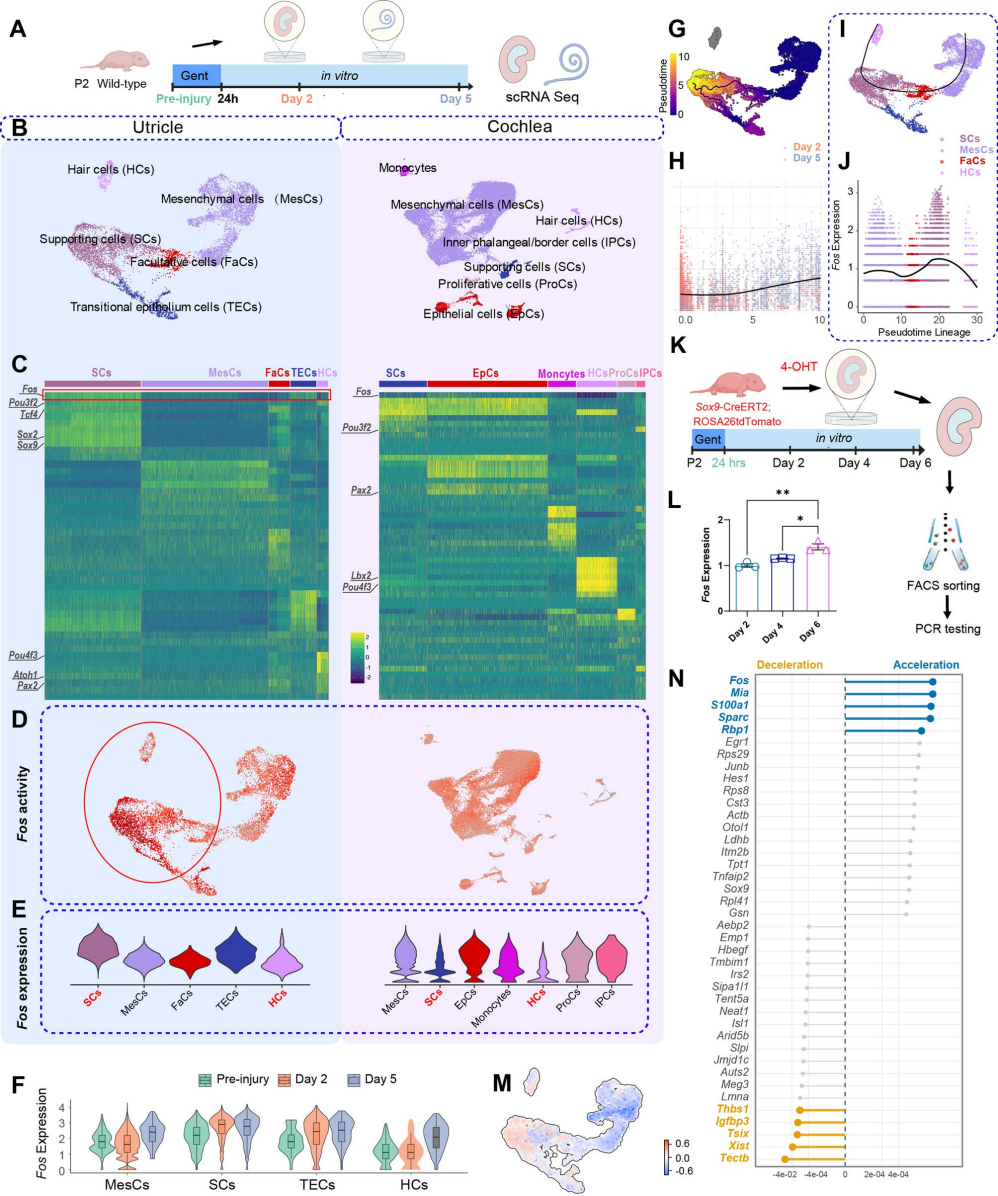
*Fos* is identified as a key regulator of inner ear regeneration by single-cell transcriptomic analysis (**A**) Utricles and cochleae were harvested from postnatal day 2 (P2) mice and cultured *ex vivo.* To induce damage, cochlear explants were treated with gentamicin for 6 h, while utricular explants were treated for 24 h. Following the injury period, samples were collected for single-cell RNA sequencing (scRNA-seq) at 2- and 5- days post-injury (B-C) Global transcriptomic landscape of inner ear regeneration. **B** UMAP (Uniform Manifold Approximation and Projection) plots of integrated scRNA-seq data from regenerating utricles and cochleae, combining the 2- and 5-day post-injury time points to visualize distinct cell clusters. **C** Heatmaps of predicted transcription factor activities (via SCENIC analysis), highlighting the top 10 active regulons for each cell cluster (**D**-**J**) *Fos* is a key transcription factor upregulated during regeneration. **D** Feature plot showing high *Fos* regulon activity (SCENIC AUC scores) projected onto the UMAP coordinates. **E**-**F** Violin plots comparing *Fos* transcript expression across different cell populations (**E**) and time points (**F**) (pre-injury control, 2-days, and 5-days post-injury in utricle explants), revealing its robust upregulation in supporting cells during utricle regeneration. **G** Pseudotime trajectory analysis of regenerating utricular cells, constructed using Monocle 3 on the integrated UMAP embeddings from 2- and 5-day post-injury utricle explants. **H** Expression profile of *Fos* along the pseudotime trajectory, revealing a sustained upregulation. **I**-**J** Trajectory inference using Slingshot confirms *Fos* as an initiator of the regenerative program. **I** Slingshot delineates the differentiation trajectory of regenerating cells. **J** Projection of *Fos* expression onto the Slingshot trajectory (**K**-**L**) qPCR validation of *Fos* upregulation in the supporting cell lineage. **K** Schematic of the experimental design for qPCR validation. Utricles from *Sox9*-CreERT2; Ai14-tdTomato (*Sox9*TM) mice were damaged with gentamicin in vitro. 4-OHT was added to the medium to label the supporting cell lineage. At 2, 4, and 6 days post-injury, tdTomato-positive cells were isolated by FACS for qPCR analysis. **L** qRT-PCR analysis of sorted tdTomato-positive cells shows that *Fos* mRNA expression progressively increases over the 6-day regeneration period (one-way ANOVA with Tukey’s post-hoc test, *n* = 3 per condition) (M-N) Dynamic modeling of *Fos* activity in the regenerating utricle. **M** RNA velocity analysis revealing a robust induction of *Fos* expression in hair cells and supporting cells. **N** Lollipop plots quantifying the top 20 genes exhibiting the highest rates of RNA acceleration and deceleration in supporting cells during utricle regeneration. *p < 0.05, **p < 0.01 Data are shown as the mean ± SEM

To investigate the molecular mechanisms underlying the differential regenerative capacities of the cochlear and the vestibular systems, we performed a single-cell comparative transcriptomic analysis of mouse cochlear and utricular tissues following ototoxic injury (Fig. 1A). Our analysis revealed marked differences in transcription factor activity, most notably in *Fos*. Using single-cell regulatory network inference and clustering (SCENIC) [18], we found robust activation of *Fos* in vestibular sensory epithelial cells, whereas *Fos* remained largely inactive in the cochlear epithelium (Figs. 1B–D). The top five core targets of this regulon, ranked by association score, were *Hes1*, *Sparc*, *Cntn1*, *Ush1c*, and *Jund*, and notably included the key hair cell fate determinants *Atoh1* and *Pou4f3*. Violin plot analysis confirmed a significant increase in *Fos* expression in the utricular sensory epithelia compared to the cochlea following injury (Fig. 1E). Furthermore, temporal analysis of the regenerating utricle indicated that *Fos* expression progressively increased along the pseudotime trajectory, corresponding with the period of cellular reprogramming (Figs. 1F-H).

To map the regenerative lineage, Slingshot analysis reconstructed the trans-differentiation trajectory from supporting cells to hair cells (Fig. 1I). *Fos* expression was highest at the onset of this trajectory within the initial supporting cell progenitors and was subsequently downregulated along the path toward the hair cell fate (Fig. 1J), reinforcing its role as a transient initiator of regeneration. To validate these dynamics in the source cell population, we cultured neonatal utricles from *Sox9*-CreERT2; Ai14-tdTomato (hereafter referred to as *Sox9*TM) reporter mice. Following the induction of ototoxic damage, we activated Cre recombinase with tamoxifen. Subsequent isolation of these newly-labeled tdTomato^+^ cells and quantitative real-time reverse transcription PCR (qRT-PCR) analysis confirmed that *Fos* transcripts were progressively upregulated (Figs. 1K-L).

Using RNA velocity analysis, we observed that *Fos* exhibited significantly increased transcriptional velocity in utricular sensory epithelial cells—including both hair cells and supporting cells—indicating its active upregulation during regeneration. Complementary analysis with the Dynamo framework [19] revealed that supporting cells displayed the highest RNA acceleration for *Fos* (Figs. 1M-N), implicating *Fos* as a key regulator of cell fate decisions during regeneration.

**Fig. 2:**
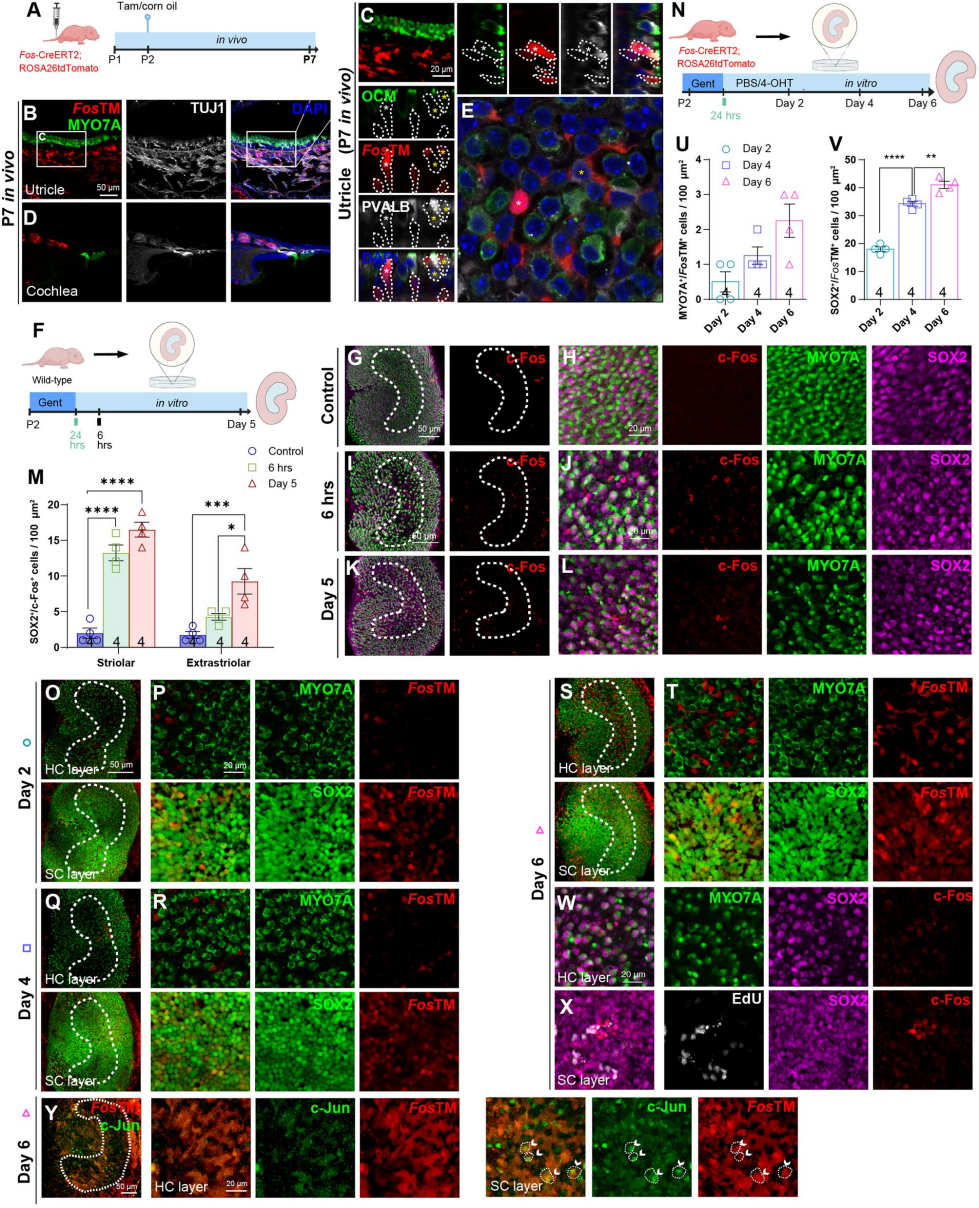
Temporal and spatial analysis of ***Fos*** expression in developing and injured inner ear sensory epithelia (**A**-**E**) In vivo analysis of *Fos* expression during postnatal inner ear development. **A** Schematic of the in vivo fate-mapping design. Tamoxifen (Tam) was administered at P2 to *Fos*-CreERT2; Ai14-tdTomato mice (*Fos*TM), with tissue collection at P7. **B**-**D** Representative images of P7 tissues showing *Fos*-driven tdTomato expression (*Fos*TM, red). **B** Low-magnification view of utricular sections. **C** Magnified view of the utricle from (**B**), showing *Fos*TM co-expression with the hair cell marker MYO7A (green). **D** View of the cochlea sections showing *Fos*TM co-expression with the neuronal marker TUJ1 (white). **E** High-magnification of the utricle striolar region showing co-labeling of *Fos*TM with PVALB (white) (**F**-**M**) c-Fos protein is upregulated in utricle explants following ototoxic injury. **F** Schematic of the ex vivo utricular explant injury model. **G**-**L** Representative images showing c-Fos protein immunofluorescence (red). **G**-**H** show the undamaged control group, while (**I**-**L**) shows gentamicin-damaged explants 6 h (**I**-**J**) and 5 days (**K**-**L**) post injury. **H**, **J**, **L** Higher magnification views of the striolar regions from (**G**, **I**, **K**), respectively. **M** Quantification of c-Fos^+^/SOX2^+^ cells in the striolar and extrastriolar regions, showing a significant increase after damage (two-way ANOVA with Bonferroni’s post-hoc test, *n* = 4 per condition) (**N**-**V**) Temporal analysis of *Fos*-lineage cells in the regenerating utricle explants using the *Fos*TM reporter line. **N** Schematic of the ex vivo utricular explant injury model. Following gentamicin (Gent) damage, explants were cultured in medium continuously supplemented with 4-OH-tamoxifen (4-OHT), with tissue collection at 2, 4, and 6 days post-injury. **O**-**T** Time-course of *Fos*TM expression after injury in the ex vivo cultured utricles. Representative images show colocalization with MYO7A (white) and SOX2 (red, supporting cells). **P**, **R**, **T** are magnified views of the striolar region. **U**-**V** Quantification of MYO7A^+^/*Fos*TM^+^ cells (**U**) and SOX2^+^/*Fos*TM^+^ cells (**V**), showing a progressive increase over 6 days post-injury (One-way ANOVA with Tukey’s HSD post-hoc test) (**W**-**X**) c-Fos acts as a transient regenerative initiator, with its expression restricted to progenitors. At day 6 post-injury, c-Fos (red) is downregulated in hair cells (MYO7A, green) (**W**), yet is expressed in the supporting cell layer (SOX2, magenta; EdU, white) (**X**) (**Y**) Co-localization of the *Fos*-lineage marker and c-Jun in regenerating cells. Representative images showing co-localization of *Fos*TM (red) and its binding partner c-Jun (green) at day 6 post-injury in the hair cell layers and supporting cell layers. White arrows indicate double-positive cells **p < 0.01, ****p < 0.0001. Data are shown as the mean ± SEM. Scale bars = 50 μm (low magnification) and 20 μm (high magnification)

To identify and track cells that express *Fos*, we employed *Fos*-CreERT2 knock-in mice crossed with the Ai14 tdTomato reporter (*Fos*-CreERT2-tdTomato, hereafter referred to as *Fos*TM) for lineage tracing. In this system, CreERT2 is driven by the endogenous *Fos* promoter, allowing for permanent tdTomato labeling of *Fos*-expressing cells upon 4-hydroxytamoxifen (4-OHT) induction. Following 4-OHT administration at P2, *Fos* expression in the cochlea at P7 was confined to spiral ganglion neurons. In contrast, vestibular epithelia showed additional *Fos*-driven tdTomato labeling in PVALB-positive Type I hair cells during the neonatal developmental stage (Figs. 2A-E, Supplementary Figs. S1A-B).

Our analysis of gentamicin-damaged auditory explants revealed a different c-Fos response between the utricle and cochlea. As early as 6 h post-injury, we observed an acute and significant increase in c-Fos^+^/SOX2^+^ cells within the utricular sensory epithelium, a response that persisted through Day 5. These cells were concentrated in the striola-a region known for its robust regenerative potential (Figs. 2F-M). Conversely, this immediate c-Fos protein expression was not detected in cochlear explants (Supplementary Figs. S1C-E).

To further validate this, we utilized the *Fos*TM lineage-tracing mouse, which confirmed this region-specific *Fos* activation. Following damage, vestibular tissues exhibited robust *Fos* activation, whereas cochlear *Fos* expression was restricted to spiral ganglion neurons (Supplementary Figs. S1C, F). Temporal analysis from P2 to P6 then showed a gradual increase in hair cells (MYO7A^+^) and supporting cells (SOX2^+^) derived from the *Fos* lineage within the utricular striola (Figs. 2N-V). Furthermore, analysis of gentamicin-damaged utricular explants revealed that c-Fos protein was prominently expressed within the supporting cell layer, where it co-localized with a subset of proliferating cells. In contrast, its expression was downregulated in the hair cell layer.

Further protein expression analysis of gentamicin-damaged utricular explants revealed a spatially distinct and transient pattern for c-Fos, consistent with its role as a regenerative initiator. The protein was expressed in the supporting cell layer, but was expressed at low levels in the hair cell layer. This pattern is consistent with its role as a transient initiator that becomes downregulated as supporting cells begin to trans-differentiate into hair cells (Figs. 2W-X).

Notably, both *Fos* lineage-traced cells and c-Jun were enriched and co-localized in the striolar epithelium (Fig. 2Y), suggesting that c-Fos and c-Jun may function cooperatively as AP-1 transcriptional complexes to mediate gene expression in response to ototoxic injury. Taken together, these findings suggest that ototoxic injury selectively triggers *Fos* expression in the utricle but not in the cochlea, underscoring inherent differences in the molecular pathways regulating hair cell regeneration between these two sensory systems.

### *Fos* is required for utricular hair cell regeneration and survival

**Fig. 3:**
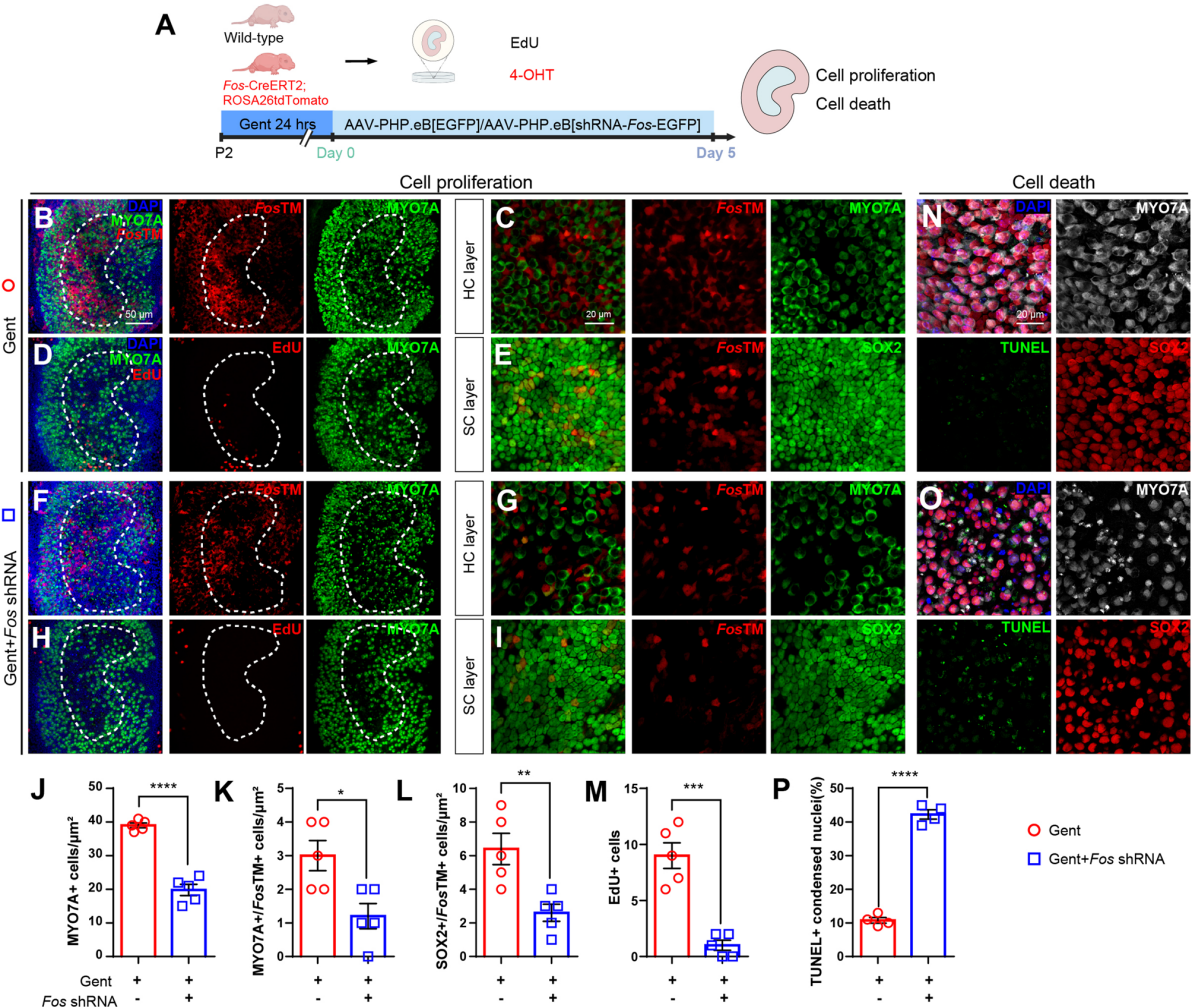
*Fos* Knockdown impairs hair cell regeneration and survival in vestibular epithelia (**A**) Schematic of the ex vivo knockdown experiment design. Utricles from P2 *Fos*TM and wild-type mouse were damaged with gentamicin (Gent), then transduced with either a control AAV (AAV-EGFP) or an AAV expressing a *Fos*-targeting shRNA (AAV-*Fos* shRNA). Explants were subsequently cultured for 5 days in medium containing both EdU and 4-OHT for the entire duration (B-I) *Fos* knockdown impairs hair cell regeneration and supporting cell maintenance. Representative confocal images of utricles treated with Control AAV (**B**-**E**) or AAV-Fos shRNA **F**-**I**. **B**, **F** Low-magnification views of the hair cell layer showing MYO7A^+^ hair cells (green) and *Fos*-lineage cells (*Fos*TM, red) in the hair cell layer. **C**, **G** Higher magnification views of the striolar region from (**B**) and (**F**), respectively. **D**, **H** Low-magnification views of the supporting cell layer showing EdU^+^ proliferating cells (red). **E**, **I** Striolar regions higher magnification views of the supporting cell layer from (**D**) and (**H**) (**J**-**M**) Quantification of cellular changes following *Fos* knockdown. Quantification of cell populations in the striolar region reveals that *Fos* knockdown significantly reduced the number of MYO7A^+^ cells (**J**), MYO7A^+^/*Fos*TM^+^ cells (**K**), SOX2^+^/*Fos*TM^+^ supporting cells (**L**), and total EdU^+^ proliferating cells in the striolar region (**M**) compared to control AAV group (unpaired Student’s t-tests, *n* = 5 per condition) (**N**-**P**) TUNEL assay showing cell death in the Gent-control (AAV-EGFP) (**N**) and AAV-*Fos* shRNA-transduced groups (**O**) with DAPI, MYO7A, TUNEL, and SOX2 labeling. Quantification of TUNEL^+^ condensed nuclei in (**P**) showed significantly increased cell death in the AAV-*Fos* shRNA-transduced group (unpaired Student’s *t*-tests, *n* = 4 per condition) *p < 0.05, **p < 0.01, ***p < 0.001, ****p < 0.0001. Data are shown as the mean ± SEM. Scale bars = 50 μm (low magnification) and 20 μm (high magnification)

To investigate the role of *Fos* in initiating hair cell regeneration following injury, we employed an adeno-associated virus (AAV)-mediated shRNA knockdown strategy (AAV-*Fos* shRNA) in gentamicin-damaged utricular explants (Fig. 3A). To validate the efficacy of this knockdown strategy, we observed its effect in *Fos*TM reporter mice. This approach resulted in a marked decrease in the number of tdTomato-positive cells observed across the sensory epithelium (Figs. 3B, F). qRT-PCR revealed a significant decrease in *Fos* transcripts, and correspondingly, c-Fos protein levels were shown to be substantially reduced by both Western blot and immunofluorescence analyses.

Following gentamicin-induced damage, *Fos* knockdown significantly compromised the regenerative capacity of the utricular epithelium (Figs. 3B-M). Quantitative assessments revealed substantial decreases in both MYO7A⁺/*Fos*TM⁺ cells and SOX2⁺/*Fos*TM⁺ cells (Figs. 3K-L). These findings suggest that *Fos* is critical not only for the generation of new hair cells but also for the maintenance of supporting cells, which serve as progenitors for trans-differentiation. Furthermore, EdU incorporation assays demonstrated that *Fos* knockdown suppressed cell proliferation in damaged explants (Figs. 3D, H, M), while TUNEL assays revealed an increase in apoptosis as indicated by an elevated number of TUNEL⁺/DAPI⁺ nuclear fragments (Figs. 3N-P).

Taken together, these findings demonstrate that *Fos* knockdown hinders both the proliferation of progenitor cells and the trans-differentiation processes that are essential for hair cell formation, while concurrently promoting apoptotic cell death.

### *Fos* overexpression promotes utricular hair cell regeneration post-injury

**Fig. 4:**
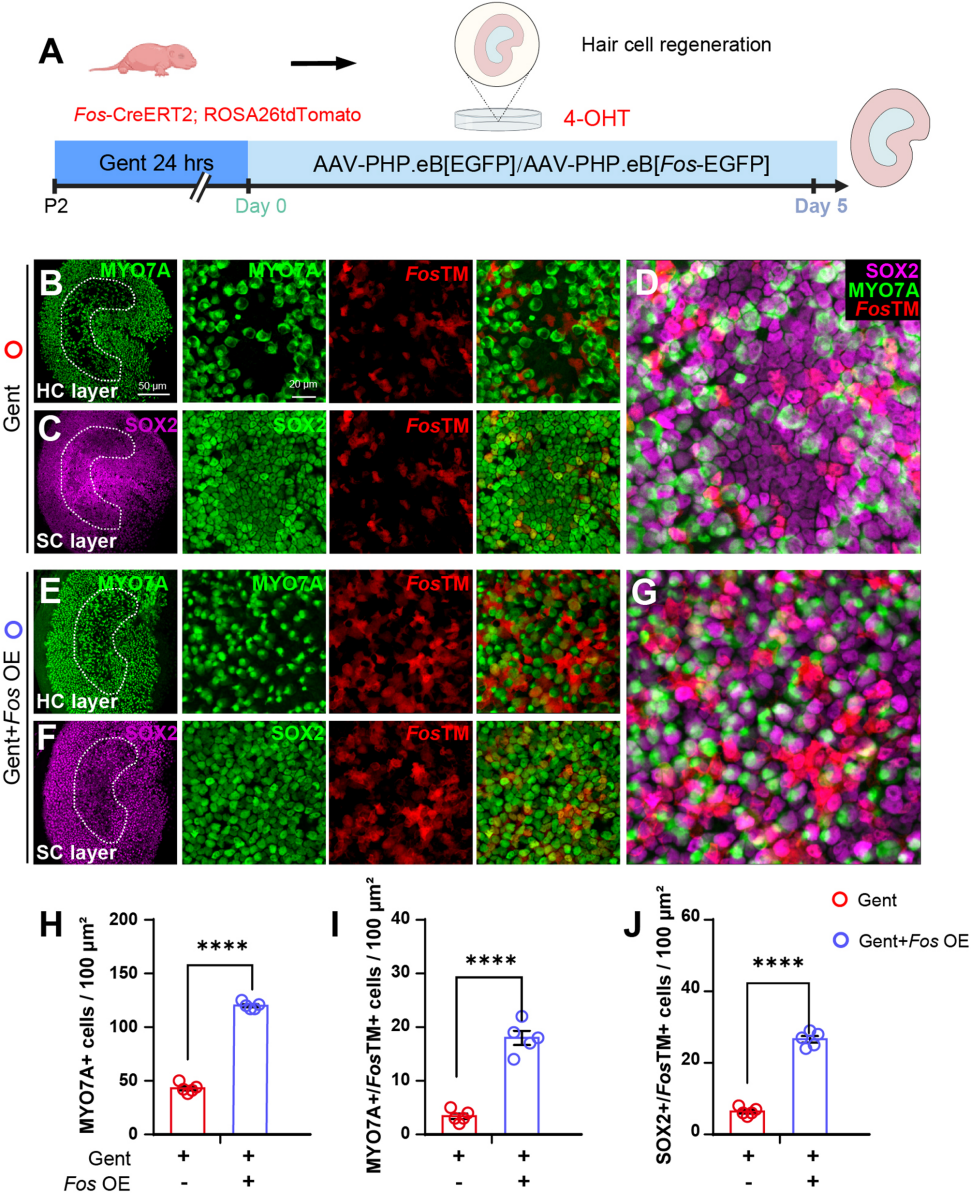
AAV-mediated *Fos* overexpression enhances hair cell regeneration in the vestibular epithelia (**A**) Schematic of the ex vivo overexpression experiment. Utricles from P2 *Fos*TM mice were damaged with gentamicin (Gent), then transduced with either a control AAV (AAV-EGFP) or an AAV expressing *Fos* (AAV-*Fos* OE). For the entire 5-day culture period, the medium was supplemented with EdU and 4-OHT (B-G) Representative confocal images of utricles treated with Control AAV (**B**-**D**) or AAV-Fos OE **E**-**G**. Images show co-labeling of *Fos*-lineage cells (*Fos*TM, red) with MYO7A (hair cells, white) and SOX2 (supporting cells, green), presented as separate projections of the hair cell and supporting cell layers. **D**, **G** Higher magnification images of the striolar region (**H**-**J**) Quantification of cellular changes following *Fos* overexpression. Bar plots quantifying the significant increase in the number of total MYO7A^+^ cells (**H**), MYO7A^+^/*Fos*TM^+^ hair cells (**I**), and SOX2^+^/*Fos*TM^+^ supporting cells (**J**) in the AAV-*Fos* OE group compared to control AAV group (unpaired Student’s *t*-tests, *n* = 4 per condition) ****p < 0.0001. Data are shown as the mean ± SEM. Scale bars = 50 μm (low magnification) and 20 μm (high magnification)

To assess whether *Fos* activation is sufficient to promote hair cell regeneration, we transduced the sensory epithelium of gentamicin-damaged utricular explants with AAV-PHP.eB vectors carrying either a *Fos* expression plasmid (AAV-*Fos* OE) or a control AAV-CMV-EGFP construct (Fig. 4A, Supplementary Fig. S2). The overexpression was confirmed at both the transcript and protein levels, with qPCR detecting upregulated *Fos* transcripts (Supplementary Fig. S2L), and both Western blot and immunofluorescence showing increased c-Fos protein (Supplementary Figs. S2M-R). In *Fos*TM reporter mice, AAV-*Fos* OE transduction led to a markedly enhanced number of tdTomato-positive cells in both the hair cell and supporting cell layers (Figs. 4B-J). Quantitative analysis demonstrated that *Fos* overexpression significantly improved utricular regeneration, with increased numbers of MYO7A^+^/*Fos*TM^+^ cells and SOX2^+^/*Fos*TM^+^ cells compared to controls (Figs. 4H-J).

**Fig. 5:**
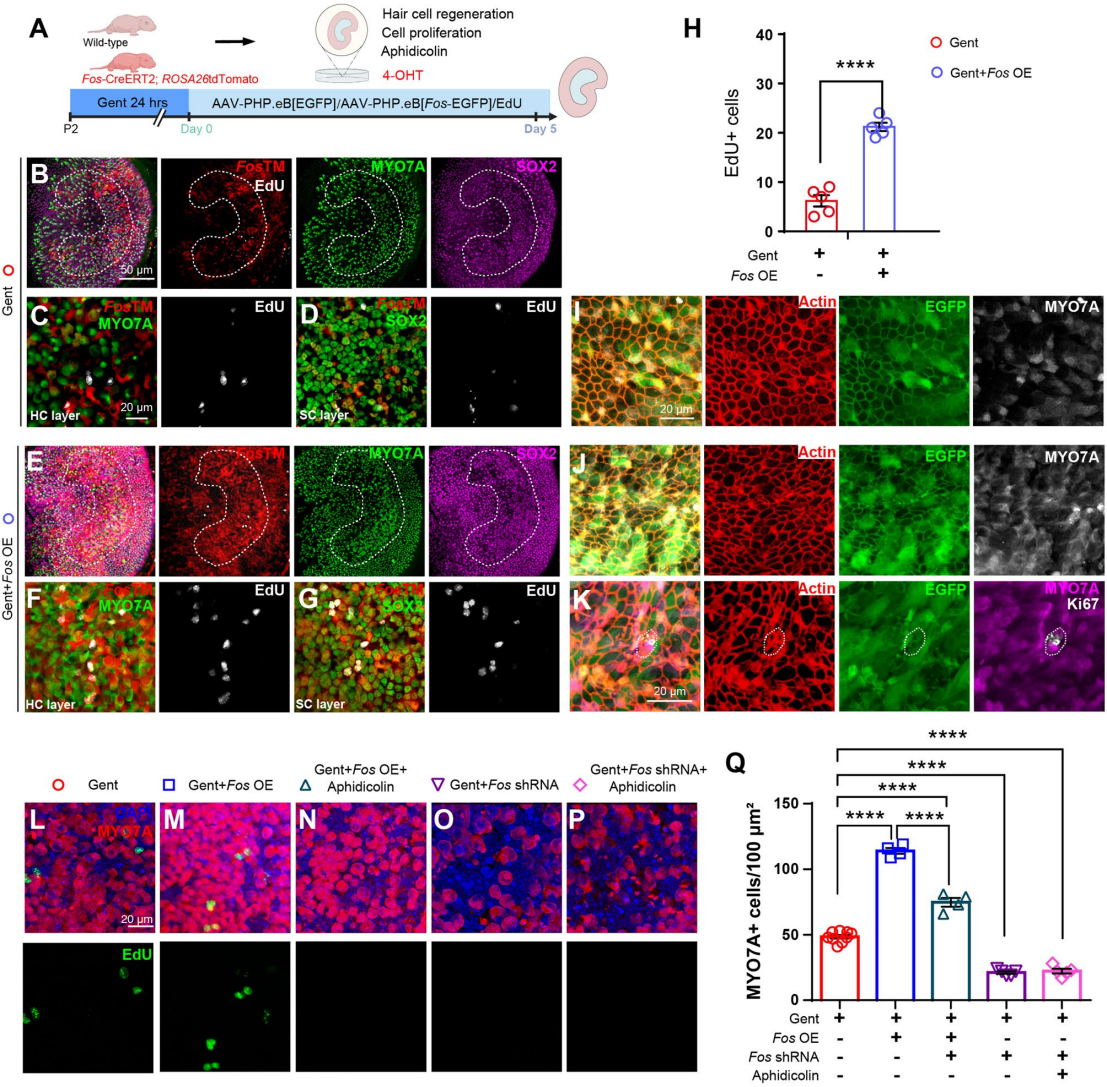
*Fos* regulon activity following AAV-mediated overexpression induces the proliferation of utricular sensory epithelial cells (**A**) Schematic of the *ex vivo Fos* overexpression and proliferation-inhibition experiment. Utricles from *Fos*TM mice and wild-type mouse were damaged with gentamicin, then transduced with control AAV (AAV-EGFP), AAV-*Fos* OE, or AAV-*Fos* shRNA, with or without the cell cycle inhibitor aphidicolin. Explants were cultured for 5 days. For the entire period, the medium contained EdU and 4-OHT. For the inhibition groups, the cell cycle inhibitor aphidicolin was also added for the entire 5-day culture duration (**B**-**H**) *Fos* overexpression significantly increases the number of proliferating cells. **B**-**G** Representative confocal images of utricles treated with Control AAV (**B**-**D**) or AAV-*Fos* OE (**E**-**G**), stained for EdU (white), *Fos*-lineage cells (*Fos*TM, red), and hair cells (MYO7A, green). **C**, **F** Higher magnification views of the striolar region from (**B**) and (**E**) respectively, focusing on the hair cell layer. **D**, **G** Higher magnification views of the striolar region from (**B**) and (**E**) respectively, focusing on the supporting cell layer. **H** Quantification confirms a significant increase in the total number of EdU^+^ proliferating cells within the striolar region compared to the control group (unpaired Student’s t-tests, *n* = 4 per condition) (I-K) Characterization of transduced and regenerating cells. **I**-**J** Images showing transduction patterns of control AAV-EGFP (**I**) and AAV-*Fos* OE (**J**) within gentamicin-damaged utricular sensory epithelia co-labeled with phalloidin (F-actin) and MYO7A. **K** A high-magnification image showing a Ki67-positive (white, proliferation marker) cell that has been transduced with *Fos*-EGFP (green) and is located in the hair cell layer (MYO7A, red) (**L**-**Q**) The pro-regenerative effect of *Fos* is dependent on cell cycle entry. **L**-**P** Representative images of utricles from five conditions: Control (**L**), AAV-*Fos* OE (**M**), AAV-*Fos* OE + aphidicolin (**N**), AAV-*Fos* shRNA (**O**), and AAV-*Fos* shRNA + aphidicolin **P**. **Q** Quantification of MYO7A^+^ hair cells across these groups. The increase in hair cells induced by AAV-*Fos* OE is blocked by the cell cycle inhibitor aphidicolin, confirming that the regenerative effect is proliferation-dependent (one-way ANOVA with Tukey’s HSD post-hoc test, *n* = 4 per condition) ****p < 0.0001. Data are shown as the mean ± SEM. Scale bars = 50 μm (low magnification) and 20 μm (high magnification)

EdU incorporation assays revealed that *Fos* overexpression enhanced proliferation in the regenerating epithelium (Figs. 5A-H). Specifically, we observed MYO7A^+^/*Fos*TM^+^/EdU^+^ cells (Fig. 5F-G), indicating new hair cells derived from dividing progenitors, and SOX2^+^/EdU^+^ cells, confirming supporting cells proliferation (Fig. 5G). Immunofluorescence analysis identified rare but significant F-actin^+^/Ki67^+^/EGFP^+^/MYO7A^+^ cells alongside abundant F-actin^+^/EGFP^+^/MYO7A^+^ cells (Figs. 5I-K), collectively demonstrating robust proliferation and successful hair cell regeneration in *Fos*-enriched regions.

To determine whether *Fos* overexpression could confer regenerative capacity to the typically non-regenerative cochlea, we similarly transduced cochlear explants with AAV-*Fos* OE using AAV-CMV-EGFP as the control. Despite robust transgene expression, *Fos* overexpression in cochlear explants did not lead to a significant increase in hair cell counts or morphological recovery compared to controls (Supplementary Fig. S3), indicating that the regenerative effect of *Fos* is context-dependent and appears specific to the utricle.

To further investigate the role of *Fos* in cell proliferation during hair cell regeneration, we treated gentamicin-damaged utricles with aphidicolin, a DNA polymerase inhibitor that blocks cell division. Aphidicolin significantly reduced MYO7A^+^ hair cells in *Fos*-overexpressing utricles. In contrast, *Fos*-knockdown utricles, which already have fewer MYO7A^+^ cells, showed no further reduction with aphidicolin (Figs. 5L-Q). This suggests that *Fos* is indispensable for cell proliferation during hair cell regeneration in the utricle.

### c-Fos directly regulates *Atoh1* to drive vestibular hair cell regeneration

**Fig. 6:**
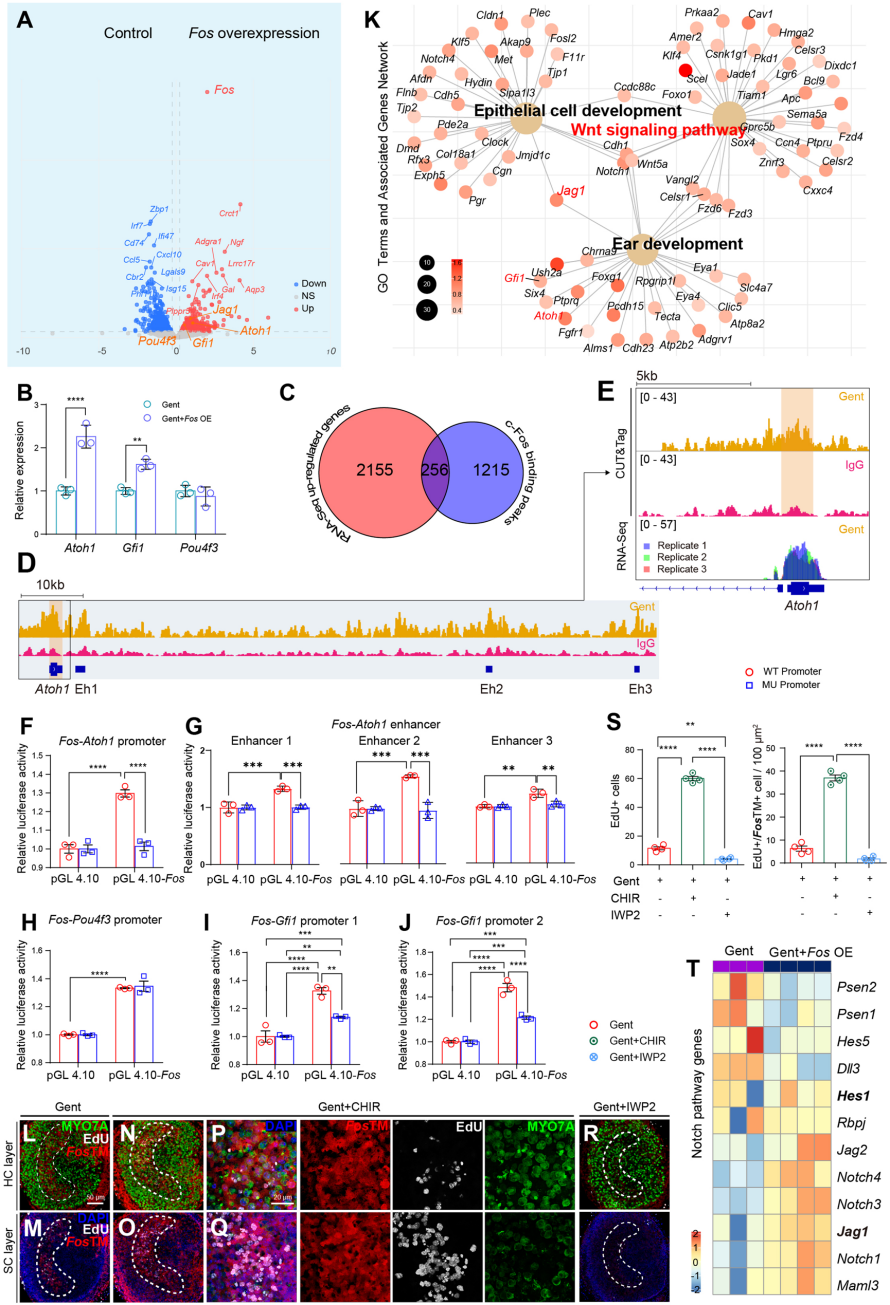
Molecular mechanisms of c-Fos–mediated regeneration (**A**-**J**) *Fos* directly binds to and activates key hair cell transcription factors. **A** Volcano plot showing differentially expressed genes between Gent-control and AAV-*Fos* OE-transduced utricular explants at day 5 post-injury. Red dots: upregulated genes (log2FC > 0.25, adjusted *p* < 0.01); blue dots: downregulated genes (log2FC < − 0.25, adjusted *p* < 0.01); gray dots: non-significant genes. **B** qRT-PCR validation of *Atoh1*, *Gfi1*, and *Pou4f3* expression levels in control versus AAV-*Fos* OE-transduced samples (unpaired Student’s *t*-tests, *n* = 3 per condition). **C** Venn diagram illustrating the overlap between *Fos* binding peaks (from CUT&Tag analysis) and significantly upregulated genes in AAV-*Fos* OE-transduced samples. **D** Integrative Genomics Viewer (IGV) track showing *Fos* binding peaks at the *Atoh1* promoter and three known enhancer regions. **E** IGV track showing *Fos* binding peaks and RNA-Seq read coverage at the *Atoh1* gene locus. **F**-**J** Luciferase reporter assays confirming direct transcriptional activation of the *Atoh1* promoter (**F**), known Atoh1 enhancer (**G**), *Pou4f3* (**H**), and *Gfi1* (**I**-**J**) promoters by *Fos* (two-way ANOVA with Bonferroni’s post-hoc test, *n* = 3 per condition). Mutation of predicted *Fos*-binding sites in the *Atoh1* promoter reduced luciferase activity to baseline levels, confirming the specificity of *Fos* regulation (**K**) Pathway-level analysis of *Fos* overexpression. Network analysis showing relationships between enriched GO terms and associated up-regulated genes in the AAV-*Fos* OE-transduced group compared to control AAV group (**L**-**S**) Crosstalk between *Fos* and Wnt signaling in regulating proliferation. Confocal images showing the effects of Wnt regulation on gentamicin-damaged utricular explants, treated with the Wnt agonist CHIR99021 or the Wnt antagonist IWP2 for 5 days in culture. (L-M) Gent-control, (N-Q) CHIR99021 treatment, (R) IWP2 treatment. Tissues labeled with EdU, MYO7A, and *Fos*TM with separate panels showing the hair cell layer and supporting cell layer. (**P**-**Q**) Higher magnification views of the striolar regions from **N**-**O**. **S** Quantification of EdU^+^ cells and EdU^+^/*Fos*TM^+^ cells across different Wnt regulation conditions (one-way ANOVA with Tukey’s HSD post-hoc test, *n* = 4 per condition). CHIR99021-mediated Wnt activation significantly increased EdU^+^ proliferating cells and EdU^+^/*Fos*TM^+^ cells. IWP2-mediated Wnt inhibition reversed the effects of CHIR99021 (**T**) Heatmap showing the differential expression of Notch pathway genes between Gent-control and AAV-*Fos* OE transductions. The color scale represents the log2FC **p < 0.01, ***p < 0.001, ****p < 0.0001. Data are shown as the mean ± SEM

To elucidate the molecular mechanisms by which c-Fos initiates hair cell regeneration post-injury, we performed transcriptomic and genomic analyses on AAV-*Fos* OE-treated vestibular sensory epithelia. RNA sequencing of these samples identified 1,504 differentially expressed genes (683 upregulated, 821 downregulated) compared to damaged controls. Notably, key hair cell fate determinants such as *Atoh1* and *Gfi1* were significantly upregulated (Fig. 6A), a finding further validated by qRT-PCR (Fig. 6B).

To determine whether these changes represent direct regulation by c-Fos rather than secondary effects, we performed CUT&Tag analysis on gentamicin-damaged utricular explants. Venn diagram comparisons revealed significant overlap between c-Fos binding peaks and the upregulated genes (Fig. 6C), with robust c-Fos binding at the *Atoh1* promoter and its known enhancer sites, indicating direct transcriptional regulation (Figs. 6D-E). Dual-luciferase reporter assays further confirmed this direct regulation. c-Fos overexpression significantly enhanced the activity of both the *Atoh1* promoter and multiple known *Atoh1* enhancers [11, 20]; critically, this effect was abolished upon mutation of the predicted c-Fos binding sites in all constructs, confirming specific activation (Figs. 6F-G). In contrast to this strong activation, *Pou4f3* displayed neither significant expression changes nor evidence of direct c-Fos regulation in our assays (Fig. 6H). Lastly, while c-Fos modestly activated the *Gfi1* promoter, the persistence of activity following canonical binding site mutations suggests that additional, indirect regulatory mechanisms likely contribute to its regulation (Figs. 6I-J). Together, these results suggest that c-Fos primarily affects early stages of hair cell fate determination by targeting *Atoh1*, rather than later maturation processes.

Gene Ontology (GO) analysis of upregulated genes revealed significant enrichment in regeneration-associated pathways (Supplementary Fig. S4A), with Wnt signaling emerging as a central node in the regulatory network. Network analysis further illustrated an interconnected regulatory architecture linking epithelial cell development and ear development via Wnt signaling components (Fig. 6K). CUT&Tag analysis demonstrated direct c-Fos binding to promoters of key Wnt pathway genes (Supplementary Fig. S4B), thus establishing a genomic regulatory relationship between c-Fos and Wnt signaling. Pharmacological experiments using CHIR99021 (a Wnt agonist) and IWP2 (a Wnt antagonist) corroborated this functional crosstalk, and co-labeling of EdU^+^ and *Fos*TM^+^ cells was markedly influenced by the activation or inhibition of Wnt signaling (Figs. 6L-S), suggesting a bidirectional regulatory interplay during hair cell regeneration.

Further analysis revealed that *Fos* overexpression significantly alters the expression of core Notch pathway components, including receptors (*Notch1*, *Notch3*, *Notch4*), ligands (*Jag1*, *Jag2*), downstream effectors (*Hes1*, *Dll3*), and γ-secretase components (*Psen1*, *Psen2*) (Fig. 6T). Network analyses positioned *Jag1* as a central hub connecting epithelial development, Wnt signaling, and ear development pathways (Fig. 6K). Increased JAG1 protein expression was confirmed by immunostaining (Supplementary Figs. S4C-I). Moreover, functional interplay between these pathways was demonstrated in Notch inhibition experiments. Treatment with DAPT [21] (a γ-secretase inhibitor) expanded *Fos* expression throughout the utricular epithelium, especially in the striola, and this effect was reversible by co-administration of the c-Fos inhibitor T-5224 (Supplementary Fig. S5). Notably, combining *Fos* overexpression with Notch inhibition synergistically enhanced both progenitor cells reprogramming and hair cells regeneration in damaged utricles, outperforming either intervention alone (Supplementary Fig. S6).

Together, these findings indicate that c-Fos directly activates *Atoh1* transcription while modulating key regenerative pathways. Wnt and Notch signaling primarily enhance cell proliferation, whereas *Atoh1* activation combined with Notch inhibition drives the trans-differentiation of progenitor cells into hair cells. Thus, c-Fos orchestrates an early regenerative response by promoting both proliferation and the direct conversion of supporting cells, ensuring effective repair after injury.

### *Fos*-dependent hair cell regeneration in adult mice in vivo

**Fig. 7:**
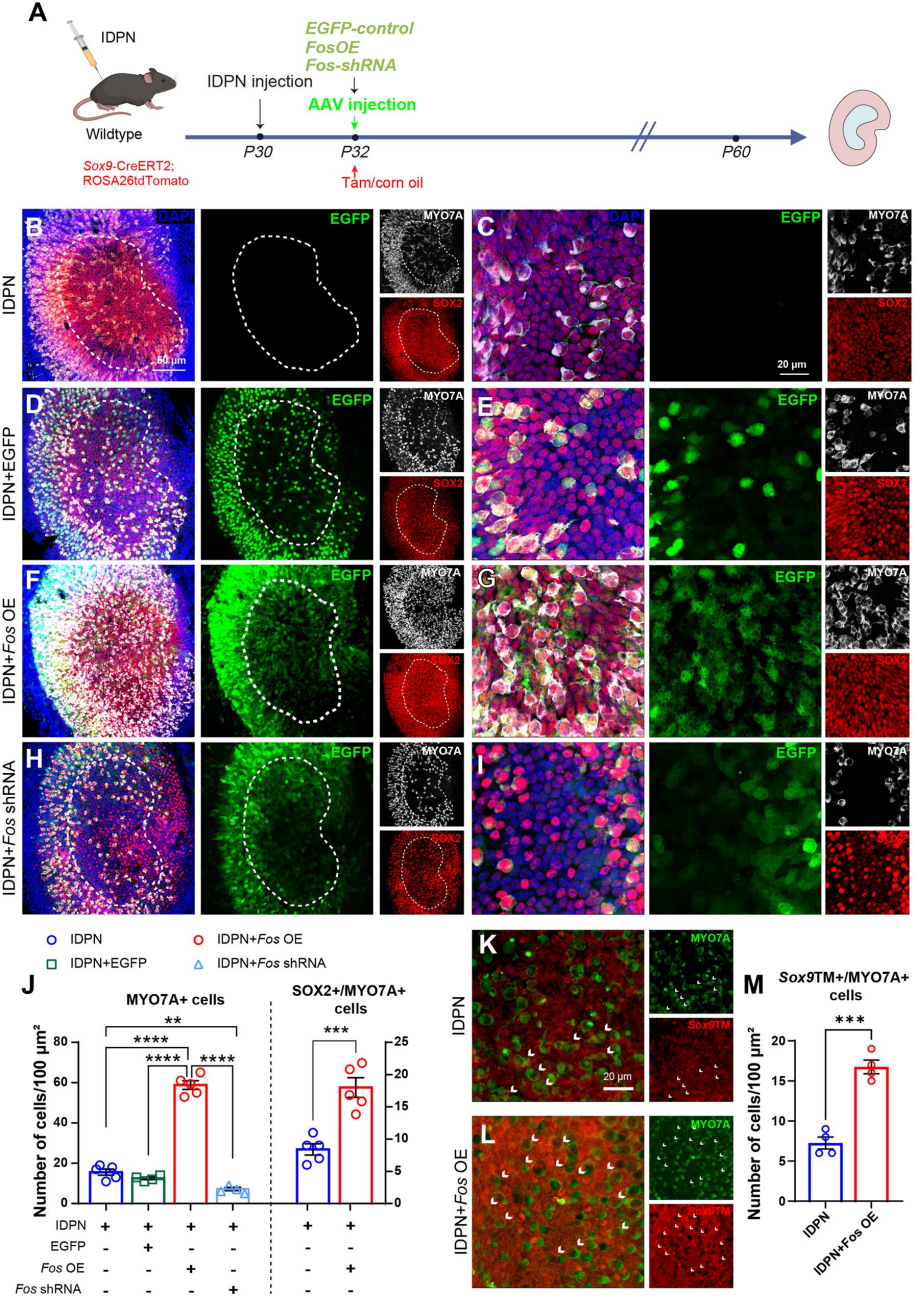
*Fos*bidirectionally regulates in vivo hair cell regeneration in adult mammals (**A**) Schematic of the in vivo experimental design. Vestibular damage was induced in adult mice (P30) via IDPN injection, followed by AAV delivery into the semicircular canal at P32. Tissues were collected at P60 for analysis. Wild-type (WT) mice were used for regeneration analysis (**B**-**J**), while *Sox9*TM mice were used for lineage tracing (**K**-**L**) following tamoxifen administration at P30-31 (**B**-**I**) Representative confocal images of adult utricular epithelia from the four treatment groups. The groups are: (**B**, **C**) IDPN-only; (**D**, **E**) IDPN + AAV-EGFP (vector control); (**F**, **G**) IDPN + AAV-*Fos* OE; and (**H**, **I**) IDPN + AAV-*Fos* shRNA. For each condition, the left panel (**B**, **D**, **F**, **H**) shows a low-magnification overview, while the right panel (**C**, **E**, **G**, **I**) presents a high-magnification view of the striolar region co-labeled for MYO7A (white), SOX2 (red), EGFP (green), and DAPI (blue) (**J**) Quantification of hair cell numbers. The left graph shows the total number of MYO7A^+^ hair cells across all four groups. The right graph shows the number of SOX2^+^/MYO7A^+^ cells (Type II hair cells). *Fos* overexpression significantly increased hair cell numbers, especially Type II hair cells, while *Fos* knockdown reduced hair cell regeneration. (Left panel: one-way ANOVA with Tukey’s HSD post-hoc test, Right panel: unpaired Student’s *t*-tests, *n* = 4 per group) (**K**-**M**) Lineage tracing demonstrates supporting cell trans-differentiation. **K**-**L** Representative images show *Sox9*TM-labeled cells (red) and MYO7A^+^ hair cells (green) in the (**K**) IDPN-only group and (**L**) IDPN + *Fos* OE group. White arrows indicate MYO7A^+^/*Sox9*TM^+^ double-positive cells, representing hair cells newly trans-differentiated from supporting cells. **M** Quantification of MYO7A^+^/*Sox9*TM^+^ cells reveals a significant increase in the *Fos* overexpression group compared to the control, confirming that *Fos* promotes the conversion of supporting cells into hair cells (unpaired Student’s *t*-test, *n* = 4 per group) **p < 0.01, ***p < 0.001, ****p < 0.0001. Data are shown as the mean ± SEM. Scale bars = 50 μm (low magnification) and 20 μm (high magnification)

To validate our ex vivo findings, we established a 3,3’-Iminodipropionitrile (IDPN)-induced vestibular injury model in P30 mice (Fig. 7A). Two separate control groups were established: an IDPN-only group that received vestibular injury but no AAV injection, and a vector-control group that received a unilateral injection of AAV-CMV-EGFP (IDPN + EGFP). By P60, quantitative analysis revealed that AAV-*Fos* OE treatment significantly increased the number of MYO7A^+^ cells (hair cells), whereas *Fos* knockdown via AAV-*Fos* shRNA impaired the net recovery of hair cells compared to controls, demonstrating the critical role of *Fos* in the net outcome of the post-injury regenerative process (Figs. 7B-J). No significant differences in hair cell density were observed when comparing the IDPN-only and IDPN + EGFP control groups, confirming that neither the surgical procedure nor AAV transduction alone affected hair cell viability or regeneration (Fig. 7B-E, J).

To identify the cellular origins of the regenerated hair cells, we used *Sox9*TM reporter mice—where *Sox9* becomes restricted to supporting cells in adult mice [22]—to trace cell lineages following vestibular damage induced at P30 (Fig. 7A). After injury and tamoxifen-induced Cre-mediated recombination, histological analysis at P60 showed an increased number of MYO7A^+^/*Sox9*TM^+^ cells in the AAV-*Fos* OE group compared with controls (Figs. 7K-M), suggesting that *Fos* overexpression promotes the trans-differentiation of supporting cells into hair cells.

Additionally, phalloidin staining showed that *Fos* overexpression improved the cytoskeletal architecture of regenerated hair cells, as evidenced by increased numbers of F-actin⁺/*Sox9*TM⁺/MYO7A⁺ cells and enhanced stereociliary bundle integrity on confocal imaging. Moreover, *Fos* overexpression enhanced neurite innervation and synaptic reconstitution, with a higher density of CTBP2 puncta and more MYO7A⁺/*Sox9*TM⁺ cells establishing connections with TUJ1⁺ neuronal fibers (Supplementary Fig. S7).

### *Fos*-mediated functional recovery after acute unilateral vestibular injury

**Fig. 8:**
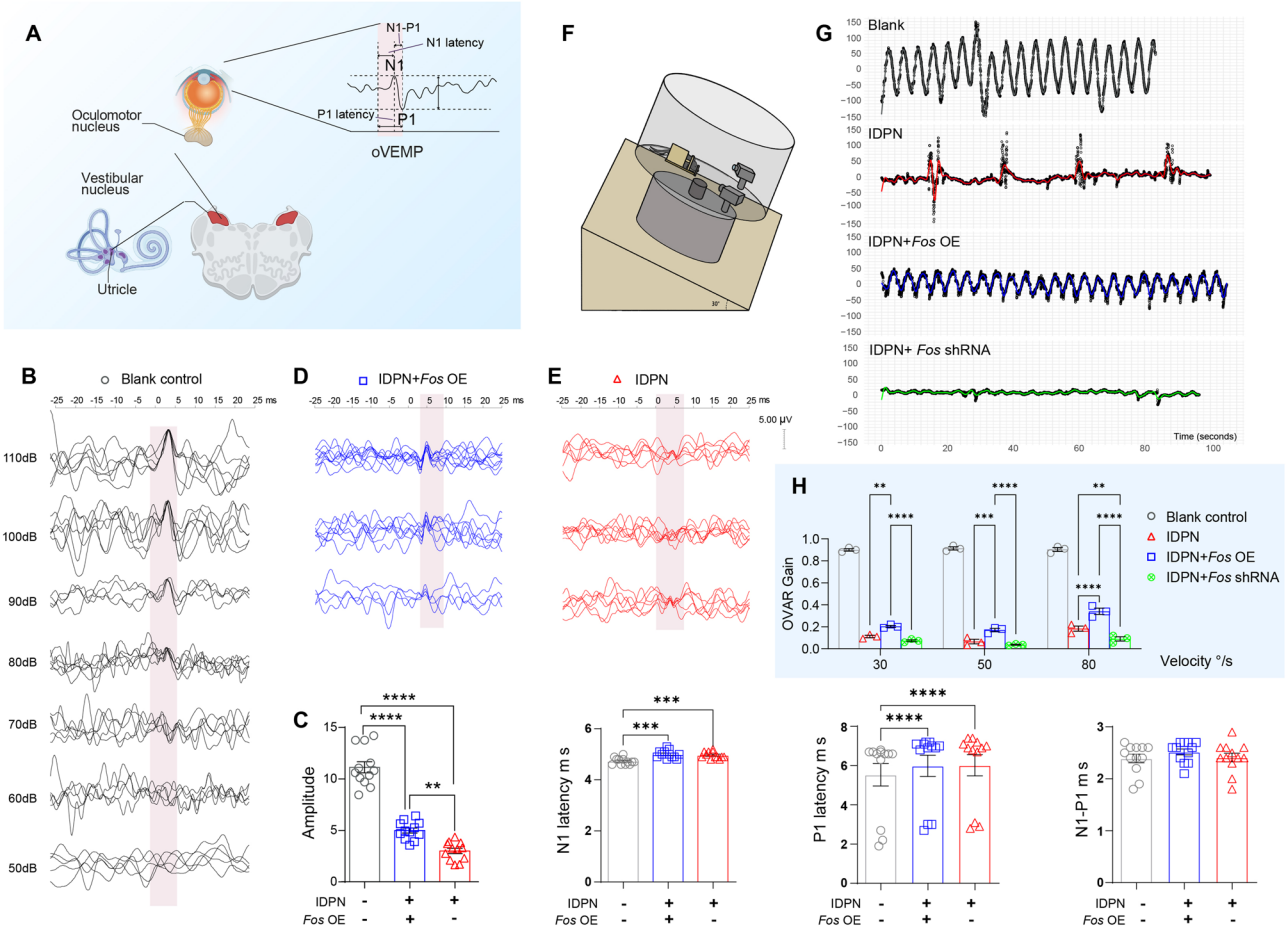
Behavioral and electrophysiological assessment of vestibular function following *Fos* modulation (**A**-**B**) Introduction to ocular vestibular evoked myogenic potential (oVEMP) analysis. **A** Schematic illustration of the oVEMP reflex pathway and corresponding waveform components. **B** Representative oVEMP waveforms recorded at threshold and suprathreshold stimulus intensities in uninjured P60 wild-type mice (**C**) Quantification of oVEMP parameters (amplitude, latencies) at 110 dB SPL across different treatment groups. AAV-*Fos* OE treatment significantly restored amplitude without affecting peak latencies compared to the IDPN control. **D**-**E** Representative oVEMP waveforms comparing an AAV-*Fos* OE-injected semicircular canal (**D**) to the IDPN control group (**E**) (F-H) *Fos* expression level bidirectionally modulates Off-Vertical Axis Rotation (OVAR) performance. **F** Schematic diagram of the OVAR testing apparatus. **G** Vector decomposition analysis of eye movement in the Blank (uninjured), IDPN-induced vestibular damage, IDPN + AAV-*Fos* OE, and IDPN + AAV-*Fos* shRNA groups. **H** Quantification of OVAR gain at varying angular velocities. *Fos* overexpression significantly improved VOR gain compared to the IDPN-damaged group, while *Fos* knockdown further impaired it. (one-way ANOVA with Tukey’s HSD post-hoc test, *n* = 3 per condition) *p < 0.05, **p < 0.01, ***p < 0.001, ****p < 0.0001. Data are shown as the mean ± SEM

To determine whether c-Fos–mediated hair cell regeneration translates into functional recovery, we conducted electrophysiological and behavioral analyses (Fig. 8A). We established three independent cohorts of IDPN-injured mice: (1) an untreated injury group (IDPN-only), which served as the functional deficit control; (2) an overexpression group (unilateral AAV-*Fos* OE); and (3) a knockdown group (unilateral AAV-*Fos* shRNA). Functional outcomes were then compared among these groups, reflecting recovery in the context of asymmetric vestibular input.

We first established baseline ocular-vestibular evoked myogenic potential (oVEMP) parameters in untreated wild-type mice at P30 (Fig. 8B). The recordings showed an N1 latency of 2.27 ± 0.29 ms, a P1 latency of 4.73 ± 0.13 ms, and a P1-N1 interpeak interval of 2.47 ± 0.24 ms, with response rates varying with stimulus intensity (Fig. 8C). Following IDPN-induced damage and subsequent treatment, ears receiving AAV-*Fos* OE displayed significant functional improvements. At 110 dB SPL, these ears exhibited higher response rates and improved P1 amplitude compared to IDPN-only controls. Although the P1 amplitude did not fully return to pre-damage levels (Figs. 8C-E), this approximately 40% recovery is a meaningful functional restoration, as it indicates successful neural innervation of a significant number of regenerated hair cells and represents a level of electrophysiological recovery known to correlate with substantial improvements in behavioral outcomes. This interpretation of a functional, hair cell-specific recovery is further supported by the fact that temporal parameters remained consistent across all groups, suggesting that the IDPN-induced damage primarily impairs hair cell function without compromising neural transmission (Fig. 8C).

Dynamic vestibular function was further assessed using off-vertical axis rotation (OVAR) testing, which quantifies the vestibulo-ocular reflex (VOR) gain and phase relationships via compensatory eye movements (Fig. 8F). AAV-*Fos* OE treatment led to velocity-dependent improvements in VOR gains, indicating enhanced dynamic ranges of vestibular processing. Conversely, *Fos* knockdown resulted in deteriorated VOR gains across natural head movement ranges (Figs. 8G-H).

In addition, behavioral analyses revealed that mice treated with AAV-*Fos* OE showed significantly improved motor coordination compared to the IDPN control group, as evidenced by lower vestibular dysfunction ratings. CatWalk gait analysis further demonstrated comprehensive locomotor improvements, including reduced stance duration and increased swing duration, indicating restored motor control efficiency (Supplementary Fig. S8).

## Methods

### Animal models and surgical procedures

All animal procedures complied with Fudan University Animal Care and Use Committee guidelines (202412030Z) and NIH standards and followed the ARRIVE guidelines. Wild-type C57BL/6J mice were sourced from Shanghai Jihui Laboratory Animal Care Co., Ltd., and Shanghai Lingchang Biotech. Transgenic lines included ROSA26tdTomato (Ai14), *Sox9*-CreERT2, and *Fos*-CreERT2 in the C57BL/6J background. Tamoxifen (0.20 mg/g body weight, Sigma-Aldrich) was administered intraperitoneally on two consecutive days at either P2-3 or P30-31 for Cre activation. Vestibular hair cell damage was induced in P30 mice via intraperitoneal IDPN injection (5 µl/g body weight). P30 mice were selected because they represent a developmental transition phase where utricular morphogenesis is complete (by P21) but progenitor-like cells persist until P35, thus providing the opportunity to investigate regeneration in near-adult systems.

For all in vivo experiments, mice were randomly assigned to their respective treatment groups using a simple randomization procedure to ensure unbiased group allocation. Furthermore, to minimize potential experimental bias, investigators responsible for performing surgical procedures, collecting tissues, and conducting all subsequent histological and functional analyses were blinded to the treatment conditions of the animals. This blinding was maintained until all data had been collected and quantified.

For in vivo virus delivery, P30 mice were randomly assigned to their respective treatment groups and anesthetized with isoflurane (0.5 L/min). All surgical procedures and injections were performed unilaterally on the left side; the contralateral right ear was left untreated. The posterior semicircular canal (PSC) fenestration approach involved a post-auricular incision on the left side to expose the left PSC approximately 3–4 mm posterior to the ear canal, using the facial nerve as a landmark. PSC lumen access was achieved using a 27-gauge needle (BD Biosciences), and access was confirmed by perilymphatic fluid leakage. AAV-PHP.eB vectors were administered using a Nanoliter Microinjection System (WPI) with a glass micropipette. The injection site was sealed with 3 M Vetbond tissue adhesive. The optimized protocol delivered 27.6 nl × 36 injections at 10-second intervals (total volume 993.6 nl), and the incision was closed using 6/0 absorbable sutures.

### AAV-PHP.eB production and purification

Three AAV-PHP.eB vectors were engineered, including AAV-CMV-*Fos*-OE (overexpression), AAV-U6-*Fos*-shRNA-CBA-EGFP (knockdown), and AAV-CMV-EGFP (control). The overexpression construct utilized a CMV promoter driving mouse *Fos* expression, while the knockdown vector incorporated U6-driven *Fos* shRNA alongside CBA-regulated EGFP reporter expression.

Vectors were produced via triple-plasmid transfection in HEK293T cells using PEI. The transfer plasmid was co-transfected with pHelper and pRep2Cap-PHP.eB plasmids. Cells were cultured in DMEM with 10% FBS at 37 °C. Viral particles were harvested after 72 h from both cell lysates and media, purified through iodixanol gradient ultracentrifugation, and concentrated by ultrafiltration. Final preparations were quantified by qRT-PCR and standardized to 2 × 10¹³ GC/mL. Vectors were manufactured by PackGene Biotech (Guangzhou, China).

Transfection efficiency was validated in gentamicin-damaged utricular explant models through EGFP immunofluorescence. The transduction rates were separately quantified via EGFP visualization in Type I, Type II hair cells and supporting cells of the sensory epithelium (Supplementary Fig. S2) [23]. Next, we confirmed the functional efficiency of the transfection. Post-transfection analysis of the utricles revealed the expected modulation of the downstream target, as confirmed by changes in c-Fos protein levels (measured by immunofluorescence and Western blot) and *Fos* transcript levels (measured by qRT-PCR). For comparison, transfection efficiency was also validated in cochlear explants following gentamicin-induced damage (Supplementary Fig. S3A).

### Establishment of the utricular ex vivo culture and damage model

Utricles and cochleae were harvested from P2 C57BL/6J mice under sterile conditions, and the temporal bones were meticulously dissected to remove the tectorial membrane and otoconia. Explants were adhered to CellTak-coated coverslips (BD Biosciences, #354240) and cultured in 4-well Petri dishes.

Explants were maintained in serum-free DMEM/F12 (1:1, Invitrogen) supplemented with N2 (1:100 dilution), B27 (1:50 dilution), and ampicillin (50 ng/ml) at 37 °C with 5% CO2. To model hair cell damage, utricular explants were treated with 2 mM gentamicin sulfate (Sigma) for 24 h, while cochlear explants received 0.5 mM gentamicin sulfate for 6 h. EdU (10 µM) was included throughout to monitor proliferation. In genetic fate mapping using *Fos*TM reporter mice, 4-OHT was applied to induce Cre recombination. For viral transduction, AAV vectors were introduced at a 1:50 dilution and maintained for 48 h.

### Single-cell RNA sequencing of utricular and cochlear explants post-gentamicin damage

Utricular and cochlear explants were harvested at 2- and 5-days post-gentamicin damage (24 explants per group) and were dissociated using Accutase at 37°C. Following centrifugation and filtration through a 40-µm strainer, cell viability was evaluated with trypan blue. Single-cell libraries were prepared using the 10X Genomics Chromium platform with the Next GEM Single Cell 3’ Kit v3.1, targeting 25,000 cells per sample.

Sequencing data were aligned to the mouse genome (GRCm39) using Cell Ranger 3.1.0, and the analysis was conducted in Seurat (v5.2.0) with SCTransform normalization. Quality control included filtering cells based on read counts and mitochondrial content, followed by principal component analysis (PCA) and uniform manifold approximation and projection (UMAP) for dimensionality reduction. Clustering was performed using FindNeighbors and FindClusters, with cell types annotated using established markers. Harmony enabled cross-timepoint integration. Transcription factor activity was inferred with SCENIC (v1.1.2), pseudotime analysis was conducted using Monocle3 (v1.3.1), and velocity vector fields, rates, and rate changes were reconstructed with dynamo-release (v1.4.1).

### Isolation of *Sox9*-lineage cells by Fluorescence-Activated cell sorting (FACS)

*Sox9*TM^+^ supporting cells were isolated from dissociated utricles of tamoxifen-induced *Sox9*TM mice at different timepoints via FACS on a CytoFLEX SRT cell sorter (Beckman Coulter). After gating to exclude debris and doublets, tdTomato^+^ cells were sorted based on their intrinsic red fluorescence, using non-fluorescent wild-type utricle cells to set the gating threshold. The purity of the sorted population was confirmed before cells were collected for downstream qRT-PCR analysis.

### RNA sequencing and analysis

Total RNA was extracted from pooled utricles (*n* = 12 per group) using an AllPrep DNA/RNA/Protein Mini kit (QIAGEN) with three biological replicates per condition. Libraries were prepared using a NEBNext Ultra RNA Library Prep Kit following standard protocols and were quality-validated on a Bioanalyzer before paired-end sequencing on a Novaseq6000.

Raw reads were processed using TrimGalore (v0.7.1), aligned to the mm39 reference genome (Ensembl release-111) using HISAT2 (v2.2.1), and quantified using featureCounts (v2.1.0). Differential expression analysis was performed using DESeq2 (v1.38.0) with adjusted *p* < 0.01 and |log2FC| >0.25 as the significance thresholds. Gene interaction networks were visualized using cnetplot in the R package enrichplot.

### CUT&Tag sequencing and analysis

We performed CUT&Tag using the Hyperactive Universal CUT&Tag Assay Kit (Vazyme, #TD904). Dissociated utricle cells (10,000 cells per sample) were bound to concanavalin A-coated beads and sequentially incubated with c-Fos antibody (Abcam) or IgG control, secondary antibody, and pA/G-Tn5 complex. Purified DNA (including *E. coli* spike-in) was processed into libraries using the Hieff NGS Tagment Index Kit. Following validation on an Agilent 4200 TapeStation, 150 bp paired-end sequencing was conducted on a NovaSeq 6000. Binding peaks were identified using MACS.

### Dual-luciferase reporter assay system

To investigate *Fos*-mediated transcriptional regulation of *Gfi1*, *Pou4f3*, and *Atoh1*, promoter fragments (2000 bp upstream of the transcription start site plus 5’-UTR) were synthesized and cloned into the pGL4.10 plasmid by Obio Technology (Shanghai, China). Additionally, DNA fragments corresponding to three previously identified murine *Atoh1* enhancers were synthesized and individually cloned. The potential *Fos* binding sites within the promoter and enhancer regions were predicted using the *JASPAR* database. Deletion mutants of these predicted binding sites were generated.

HEI-OC1 cells were seeded in 96-well plates for 24 h prior to transfection. *Fos* expression plasmid (0.1 µg) was co-transfected with each promoter reporter plasmid (0.1 µg) and pRL-TK (0.005 µg) using Lipofectamine 3000 (0.5 µL). After 48 h, cells were lysed with passive lysis buffer, and luciferase activities were measured using the Dual-Luciferase Reporter Assay System. Firefly luciferase activity was normalized to Renilla luciferase activity. All experiments were conducted in triplicate.

### Immunohistochemistry and analysis

Tissues were fixed in 4% PFA (20 min), blocked with 10% donkey serum/1% Triton X-100 (1 h, 37 °C), and incubated with primary antibodies overnight at 4 °C followed by Alexa Fluor-conjugated secondary antibodies. EdU labeling (Invitrogen, #C10337) was used to track proliferation, DAPI was used to counterstain the nuclei, and Alexa Fluor 488-phalloidin was used to visualize F-actin in stereocilia bundles.

Super-resolution imaging employed a Leica STELLARIS STED microscope. F-actin nanostructures were resolved using time-gated STED microscopy (592 nm depletion laser) with a 100×/1.4 NA oil immersion objective, achieving ~ 40 nm lateral resolution. Regions were captured using optimized depletion parameters and time-gated detection.

Cell quantification was performed using LAS X software. Statistical analyses in GraphPad Prism 10.4.1 included unpaired Student’s *t*-tests for two-group comparisons and two-way ANOVA with Tukey’s test for genotype-region interactions. Results are presented as means ± S.E.M., with significance thresholds of *p* < 0.05 (*), *p* < 0.01 (**), *p* < 0.001 (***), and *p* < 0.0001 (****).

### Ocular vestibular evoked myogenic potential (oVEMP)

Mice were fasted for 12 h before anesthesia with pentobarbital sodium (40 mg/kg, i.p.), and anesthesia was confirmed by the absence of corneal and pain reflexes. Custom 3D-printed holders secured the animals according to body weight. The eye position was stabilized using negative pressure to properly position the inferior oblique muscle.

Recording electrodes were placed subcutaneously beneath the contralateral inferior oblique muscle (3 mm below the lower eyelid), with the reference electrode at the nose tip and the ground electrode at the vertex. ER3C tubal insert earphones were positioned in the external auditory canal.

All recordings were made in an electromagnetically shielded chamber under dark, quiet conditions. EMG signals were captured using a Smart EP system with 4 ms clicks as stimuli (200 sweeps averaged per recording at 500 Hz). Signals were amplified 100,000 times and band-pass filtered (30–3000 Hz) with an 8–100 µV acceptance window. The stimulus rate was 5 Hz with a 50 ms analysis time.

Testing began at 110 dB SPL and decreased in 10 dB steps until the response disappeared. Threshold measurements were confirmed through duplicate recordings. Acoustic stimuli were delivered to the affected ear while recording from the contralateral inferior oblique muscle. Measurements were taken 30 days post-IDPN application.

### Rotarod test

Motor coordination and balance were evaluated using a rotarod test (47600-Mouse Rota-Rod, UGO BASILE, Italy). Prior to testing, mice at 4 weeks of age received training sessions where they were placed on a stationary rod (5.7 cm in diameter) that was then accelerated to 10 rpm for 10–20 s. For the test sessions, mice were placed on the rod rotating at a constant speed of 25 rpm, and their latency to fall was recorded with a cut-off time of 300 s. Tests were performed at four time points, including before IDPN application (baseline) and at 7, 14, and 30 days after IDPN application. Three trials were conducted for each mouse at each time point with 5-minute intervals between trials. The test was terminated when the mouse fell from the rod, and the time until falling was recorded.

### Vestibular dysfunction rating (VDR)

We assessed vestibular function using a comprehensive VDR system evaluating both spontaneous behaviors and anti-gravity reflexes. Spontaneous behaviors included head bobbing, circling, and retropulsion observed during 1 min monitoring periods. Anti-gravity reflexes were tested through tail-lift, air-righting, and contact inhibition of righting reflexes. Each parameter was scored from 0 (normal) to 4 (severe dysfunction), with total scores ranging from 0 to 24. Assessments were conducted at baseline and at days 7, 14, and 30 post-IDPN application.

### OVAR test and gait analysis

Vestibular function was evaluated using OVAR testing. Following anesthesia, mice were mounted on a custom rotating platform with their heads immobilized. Eye movements were tracked using infrared video-oculography during rotational stimuli at 30°/s, 50°/s, and 80°/s. The recorded eye position data underwent Fourier analysis to determine movement amplitude, with gain calculated as the ratio between response and stimulus amplitudes [24].

For gait analysis, we employed the CatWalk XT automated system. Mice traversed a glass-floored walkway in darkness, with their paw contacts illuminated through internal light reflection and captured by a high-speed camera. The system automatically detected and classified paw prints, recording parameters including position, size, and pressure intensity.

## Discussion

This study identifies c-Fos as a pivotal early responder and master regulator of hair cell regeneration in the mammalian vestibular system, orchestrating a hierarchical molecular program that links immediate-early injury responses to cell fate determination. Following ototoxic injury, c-Fos is rapidly activated and initiates a dual-mechanism response: it directly activates the transcription of the master differentiation factor *Atoh1* to drive cell fate conversion, while simultaneously modulating the Wnt and Notch pathways to control progenitor proliferation. This balanced approach ensures that the progenitor cell pool is first expanded and then efficiently guided toward a hair cell fate, a mechanism that appears unique to the vestibule and underscores the fundamental divergence in regenerative competence between the cochlear and utricular sensory epithelia.

As a member of the immediate-early gene family, c-Fos functions as a rapid molecular sensor, translating diverse stimuli—such as growth factors, stress, neurotransmitters, and synaptic activity—into long-term transcriptional adaptations. Its rapid induction positions c-Fos as a “third messenger” in signal transduction. In this study, we found that c-Fos is highly enriched in the striola of the utricle, a region with inherent regenerative competence, where it appears to switch progenitor cells into a regenerative state [25]. This spatiotemporal specificity aligns with recent observations in other regenerative systems, where activator protein-1 (AP-1) factors serve as key regulators of tissue repair and progenitor activation [26–28].

Hair cell regeneration differs markedly between the mature mammalian cochlea and vestibule, reflecting their distinct regenerative capacities. In the cochlea, spontaneous regeneration is absent, rendering hearing loss irreversible. Although *Atoh1* is essential for differentiation, in the mature cochlea, *Atoh1* alone is insufficient for robust regeneration [29]. Combined overexpression of *Atoh1*, *Gfi1*, and *Pou4f3* significantly enhances regeneration, as evidenced by mature markers (MYO7A, prestin), stereocilia, and synaptic connections, indicating that all three factors are required to overcome epigenetic and signaling barriers in cochlear supporting cells [30]. In contrast, the vestibular utricle retains limited regenerative capacity, particularly early in development. Here, *Atoh1* alone efficiently converts supporting cells into mature hair cells [31, 32], with *Pou4f3* and *Gfi1* acting as auxiliary enhancers [33]. This difference stems from distinct epigenetic landscapes: utricular supporting cells are more permissive to *Atoh1*-driven reprogramming, while cochlear cells demand all three factors [34, 35].

In this context, our study clarifies c-Fos’s upstream role in vestibular regeneration. RNA-sequencing, CUT&Tag, and dual-luciferase assays demonstrate that c-Fos directly activates *Atoh1*, partially affects *Gfi1*, and does not directly regulate *Pou4f3*. The robust, utricle-specific induction of c-Fos post-injury therefore provides the initial trigger for this permissive environment. Thus, c-Fos selectively drives vestibular regeneration by targeting key fate determinants, while its weaker effect in the cochlea underscores the organ’s unique molecular constraints.

However, the finding that c-Fos overexpression fails to induce regeneration in the cochlea is a critically important result that underscores the profound regenerative differences between the two sensory epithelia. Beyond general ‘epigenetic silencing,’ we speculate that the cochlea fosters a more active ‘inhibitory environment’ that prevents the initiation of a regenerative program. This active suppression, rather than a merely passive restrictive state, would explain why even a potent master regulator like c-Fos is insufficient to overcome the molecular barriers in the cochlea. Unlocking the regenerative potential of the cochlea, therefore, may require not only the introduction of pro-regenerative factors but also the simultaneous neutralization of these dominant inhibitory pathways.

Our study also reveals an intriguing switch in the *Fos*-lineage: while linked to Type I hair cells during development, its overexpression drives the regeneration of Type II hair cells in adults. This phenomenon may reflect either a context-dependent function, where the post-injury microenvironment alters c-Fos activity, or an intrinsic regenerative bias of adult supporting cells toward a Type II fate.

Beyond direct regulation, c-Fos synergizes with Notch and Wnt signaling [36, 37] to control regeneration. Notch maintains supporting cell quiescence [38], while its inhibition promotes trans-differentiation by de-repressing *Atoh1*[39]. c-Fos overexpression upregulates *Jag1*, a Notch ligand that boosts proliferation [40, 41], yet Notch inhibition enhances hair cell conversion. This positions c-Fos as a molecular rheostat. A key question from our knockdown studies is whether the regenerative failure is merely a consequence of induced apoptosis. However, our finding that *Fos* overexpression actively enhances proliferation—beyond simple cell protection—demonstrates a direct pro-regenerative function. Thus, c-Fos simultaneously balances three critical outcomes: pro-survival signals, proliferation (via Wnt/Notch regulation), and trans-differentiation (via *Atoh1*activation and Notch inhibition). CUT&Tag data reveal that c-Fos directly modulates Wnt targets like *Lgr5* and *Axin2*, possibly facilitating β-catenin binding [42].

Functional assessments confirm that c-Fos-mediated regeneration improves vestibular function, evidenced by enhanced oVEMP responses, VOR gains, and motor coordination in automated gait analysis. Clinically, c-Fos holds promise as a regenerative target, though challenges like targeted delivery and off-target effects remain. Localized c-Fos activators or AAV-mediated therapies with tissue-specific promoters could address these hurdles.

### Limitations and future directions

While our study underscores the therapeutic promise of c-Fos in hair cell regeneration, several limitations require attention. Our use of gentamicin-based explant models may not fully reflect the complexities of vestibular balance disorders. Moreover, the regulatory network linking c-Fos to the Notch/Wnt pathways remains partially mapped, and our 30-day endpoint analysis leaves the long-term stability and innervation of regenerated hair cells uncertain.

To overcome these challenges, future work should test c-Fos across varied damage models and age groups. Probing the full regulatory network, perhaps via single-cell chromatin accessibility profiling, could clarify whether c-Fos serves as an epigenetic pioneer or depends on cofactors for chromatin remodeling. Extending evaluations beyond 30 days would also shed light on the durability and functionality of regenerated hair cells.

Future research should explore cell type- and dimer-specific effects of c-Fos phosphorylation. Understanding how AP-1 dimers (e.g., c-Fos/c-Jun) influence DNA binding and transcriptional outcomes is critical [43]. High-throughput and single-cell approaches will help dissect dimer dynamics, post-translational modifications, and the broader transcriptional network [44, 45]. Targeting kinases like RSK2 or ERK, or modulating AP-1 dimers, could refine therapeutic strategies for hair cell regeneration.

Furthermore, a critical consideration for clinical translation is mitigating the potential risks of chronic AP-1 activation, given its links to oncogenesis, which will require strategies for precise and transient delivery. Developing spatiotemporally precise intervention systems would refine therapeutic timing for c-Fos activation. Additionally, validating these findings in human inner ear organoids from vestibular disorder patients could confirm translational potential and reveal species-specific hurdles to regeneration.

## Conclusion

This study identifies c-Fos as a central regulator of hair cell regeneration in the mammalian inner ear. We show that c-Fos enhances vestibular progenitor plasticity and hair cell differentiation by directly regulating *Atoh1* and integrating the Notch and Wnt signaling pathways. These findings establish c-Fos as a promising molecular target for regenerative therapies, offering broad implications for the treatment of vestibular balance disorders.

## Supplementary Information

Supplementary Material 1.

Supplementary Material 2.

## Data Availability

The transcriptomic and CUT&Tag data have been deposited in the Gene Expression Omnibus (GEO) database under accession numbers GSE295480 and GSE267094, respectively.
