# Supplementary material for: Ototoxicity-induced c-Fos activation underlies the regenerative capacity of the vestibular sensory epithelia
Source: Cell Commun Signal. 2025 Oct 8;23:421. doi: 10.1186/s12964-025-02446-y (PMC12506291; doi:10.1186/s12964-025-02446-y)
Supplement: Supplementary file 1 — Supplementary Material 1. [file 12964_2025_2446_MOESM1_ESM.docx]

**Experimental Design**

# Transcription Factor Gene: *Fos*

## Plasmid Construction Plan:

To generate the c-Fos overexpression vector, the mouse *Fos* coding sequence was cloned into the pcDNA3.1(+)-MCS-3xFLAG vector, resulting in pcDNA3.1(+)-*Fos*-3xFLAG. The empty vector served as a control.

# Promoter Gene

## Atoh1

### Promoter Sequence: 5'UTR upstream 2000bp + 5'UTR

>mm39_ncbiRefSeqCurated_NM_007500.5 range=chr6:64704109-64706306 5'pad=0 3'pad=0 strand=+ repeatMasking=none

accaaatgaagaacaaagccgtcatggctacctaacctttccaggctctcacgttgtctttcatacacaattgatgatgttgggtccctcggttactggaaagtcttgcttttcagattgtacttgacagttagtaaagctaggtacagtgttggttgatttcaacagtacccagaccagtctccaaaaatacagaagaatgctgtacccgccaaataaaggcattattaccttttcctttattgttgtcgatggttgtcttttaggtttggagggtacttcctgatttacctgtctaccccccaccccatctattgttagagtatttatttctgcagagcttacactctttaagaaaagggctcctctgatctttctgtcagcattggaaggcccggaactgttctttatacagtagcaaatgggcacactggaaatagagaaacaggtgaagtctgacgataactttgaactctcttctgtccttctctatcttgtgagatataaacactagtttactccagccgctgaaatccttcgcacaaggagctccagatggcaggttggatcaggtccctgctagcggaacatttcaacggcactggaccctgcaaagacaggttccagggagctttctgcggtaccatctgccggaagacgaaagtcctcctagagtagcaaccaccctgaaaatcgaaagcaccagccacagttgccacccactctggtgccccagggacgaagaaagcccaaactggagagcactaggagcaagctggggcgtgtcccgagccaggaagtttctgattggctctttgtggtaaactaccaggcggagggctaaagaggtaggaggggagcggtttatagagggagtgaacaccctaactaaacagagcgggacaggtgggtttgactgtggtttctgggcgtttgcgcaagaagccacgcgcactagtaaaccggggcctcccgaggcagcagtttcaaaatgcccagaatcacaaagaacccaaagaccttttgcactaattccggcagcctcctgtggctctgcgttta**tgagtcct**acgctgtgagttttgaaactgtttgcatgatcccttgcgatagctcagcgtagcgcgatatcctccgcgaagggcttttgcagactgagaccctaacgggctctaagaaacacgctcaaaggctcctggggcaatcccgattttggtatgtcataactataccgcagctttactttccattaaaaaaaaaaaagactacatgaaaaaaaatccaagtactatgtggaccaagagatgcagacaaagtcaaaattaagaaatacgttcccagaagcgaatggttttcaccttgttctgatttttgattagacgccacttaacacttctacaaattaaatgtgcaataggtaagagctatatacacacctctctatatgtatttctgtcacattgcacatgggcgattttacgagtttcttgagagctgtgatccaggcttggagggatctcaggccaggcgggctactccagttggtgccccgaaggctctaggcgcgtccagcttggccgggagccgcctaccctgggtctcatctgagctggggccgctaggacaccagccgggagcccgcttcttctcccctcccaccccccattcccc*tcactcag*gtcgcctgggaggtgacaccctcaggagtcgaattacagccccacaattaacatatgaatttccagaactgactagagggacaagggggaatttttttttctttttttttttttttccagagccagagctgaactcctcggcacacagtaacttttcactgggtgtaaaaactctttgattggctcctcgcacgcgcctgcccgcccccttctttgactgggcagacacgcgactggcgcaaggagggggctgggaggggagccgggggagatacactggcgcgtgccgctttttaaaggggcgcagcgccttcagcaaccGGAGAAGCTTCGTTGCACGCGACCTGGTGTGCGATCTCCGAGTGAGAGGGGGAGGGTCAGAGGAGGAAGGAAAAAAAATCAGACCTTGCAGAAGAGACTAGGAAGGTTTTTGTTGTTGTTGTTCGGGGCTTATCCCCTTCGTTGAACTGGGTTGCCAGCACCTCCTCTAACACGGCACCTCCGAGCCATTGCAGTGCG

Construction Synthesis Length: 2198bp

### Binding Site Prediction Results:

MA0099.1.Fos::Jun (excluding repeated sites, predicted targets marked in sequence)

| Matrix ID | Name | Score | Relative score | Sequence ID | Start | End | Strand | Predicted sequence |
| --- | --- | --- | --- | --- | --- | --- | --- | --- |
| MA0099.1 | MA0099.1.Fos::Jun | 7.347607 | 0.853757 | mm39_ncbiRefSeqCurated_NM_007500.5 | 1497 | 1504 | + | gtgatcca |
| MA0099.1 | MA0099.1.Fos::Jun | 7.269981 | 0.851186 | mm39_ncbiRefSeqCurated_NM_007500.5 | 998 | 1005 | - | gtgattct |
| MA0099.1 | MA0099.1.Fos::Jun | 6.642602 | 0.830412 | mm39_ncbiRefSeqCurated_NM_007500.5 | 130 | 137 | + | gttagtaa |
| MA0099.1 | MA0099.1.Fos::Jun | 6.529458 | 0.826666 | mm39_ncbiRefSeqCurated_NM_007500.5 | 1062 | 1069 | + | atgagtcc |
| MA0099.1 | MA0099.1.Fos::Jun | 5.980823 | 0.8085 | mm39_ncbiRefSeqCurated_NM_007500.5 | 1671 | 1678 | + | ctcactca |

gtgattct reverse complement: agaatcac

MA0099.2.FOS::JUN (excluding overlapping targets, 2 targets marked in sequence)

| Matrix ID | Name | Score | Relative score | Sequence ID | Start | End | Strand | Predicted sequence |
| --- | --- | --- | --- | --- | --- | --- | --- | --- |
| MA0099.2 | MA0099.2.FOS::JUN | 6.789232 | 0.858566 | mm39_ncbiRefSeqCurated_NM_007500.5 | 376 | 382 | - | tgacaga |
| MA0099.2 | MA0099.2.FOS::JUN | 6.789232 | 0.858566 | mm39_ncbiRefSeqCurated_NM_007500.5 | 1452 | 1458 | - | tgacaga |

tgacaga reverse complement: tctgtca

tgacaga reverse complement: tctgtca

### Mutation Strategy:

All 7 sites will undergo deletion mutation

### Construction Plan:

Vector: H352 pGL4.10

Wild-type promoter construction: pGL4.10-*Atoh1* promoter(WT)

Mutant promoter construction: pGL4.10-*Atoh1* promoter(MUT)

Control: H352 pGL4.10

### Dual Luciferase Assay Groups:

1. pGL4.10 + pcDNA3.1(+)-MCS-3xFLAG + Renilla plasmid co-transfection in HEI-OC1
2. pGL4.10 + pcDNA3.1(+)-*Fos*-3xFLAG + Renilla plasmid co-transfection in HEI-OC1
3. pGL4.10-*Atoh1* promoter(WT) + pcDNA3.1(+)-MCS-3xFLAG + Renilla plasmid co-transfection in HEI-OC1
4. pGL4.10-*Atoh1* promoter(WT) + pcDNA3.1(+)-*Fos*-3xFLAG + Renilla plasmid co-transfection in HEI-OC1
5. pGL4.10-*Atoh1* promoter(MUT) + pcDNA3.1(+)-MCS-3xFLAG + Renilla plasmid co-transfection in HEI-OC1
6. pGL4.10-*Atoh1* promoter(MUT) + pcDNA3.1(+)-*Fos*-3xFLAG + Renilla plasmid co-transfection in HEI-OC1

## Pou4f3

### Promoter Sequence: 5'UTR upstream 2000bp + 5'UTR

>mm39_ncbiRefSeqCurated_NM_138945.2 range=chr18:42525662-42527707 5'pad=0 3'pad=0 strand=+ repeatMasking=none

agaccacatccctggagagactgcaggctgttctttaggttaaaaaggaggtgctaacatttgttccatcctttcaacatgttggatgccgtttcaagtgcttaccaccaagtaaccctcactacagtaggggaaactactgtatgcatctgtatacagatgggcactgcagtatagaggcctcaagatgactgtctcctggcctctcagccagtagaagtagaagcagcagtgtgctctgagtctgatggcagcatgcactgggttgtgaagaagtgtcctgagtgcacagtgaggtcatcatcatcatccttctcctccagcaagacccaagaaagctacctgagtaccatttcagttggcctccctcacagattgatgattgattgattgattgattgttgttgttgttttgccacagcaaggaaggacctagaatgtgttttgggatgagttgagaaaggactggctggcagtcctgaggaaagagggggagccaggtcagtgtagggtgtcatcttagactaaattcatccctgggtcctagactttccacactctgccccctcagtgttaagattctgggaaacccagcactcttgagcacagagacagtgctgcaggacacagctgggcaggaagaatttctcttccaacccgaaggcagctctccagccggccacagtcccaggattccccatccattattcatggtgtttattagctcccaggaagggagaggaaagaaggagaggaggcgctagggaaataggggacacagaggatggggcagagacaaacttaggacccttaagttgggggaacttctaaaggaagaagaggaggctaagaggtgaagagcccaaagtcagacatggtgttggctccaaaccagtga**gtgaatgac**actgctcacaggtaggagatcttgggctaacttatttggctcttgtttcctttggctgtctaatgggatagcagtagctactctgtagtgtcctgtaggaacgttggtcctatacactaacaatactttttaaatattgagtgctcaatacttttctcattatgttattattattattattattattattattattattattattatcattgcctcgcacaaatcagtagctcaaaaatgatagctgttgttacaggtattggggctgctaagctctacagaagtatgcagaagaattgctccagctaggccagctgtcctctactgttccttagcaagctttctggggggtgggggggaaagggagaacttggcccacaagttctgtttctcagatgaaagctgaaacagccagggttaggaagaagccacaggcttgtggatcaggaagcctttcttccacccaagctcacagaggggagtatcacccgataagcattctggtaacctctggtccttgcccaggctggtttctgagtttggaggcaccttttcgctatgcactgggatgcaggctttgagcttcagactcagagcaccttgaaaatagccagtttggggtgtgtgtgtgtgtgtgtgtgtgtgtgtgtgtgtgtgtgtg*tgtctcag*gagcttaagtctgccctttcatccaggcagctcgagctgggacaggacagaggtttaggactttatgcaaagaagtccaggaggaagaagaaagaatctgtaaagtctggcaagctggagccaggtggggcggggcggacaggaagaggccctgccaggccggggtataaatgctgtggaggggggcggccgcatcaggctcagagtggcgcgccgagacctgcggtcccgccttgcctcccgggccgcccctgcgagtcccgggcgcgtgtgcacgtctgcgcgtgcccgggcccttcctggcagactgcttgtaagatgagtgaagaagcaggtgggggagaggggaggcagcaagcgagagggcgaggggagcgctggcgctgagcggcgctcacttggagcgcggagagctagcaagacgAGCTTGATTCCATGTCCCCCGCTGCCTCCCTGCCAGACTCCCGAAG

Construction Synthesis Length: 2046bp

### Binding Site Prediction Results:

MA0099.1.Fos::Jun (excluding overlapping sites):

| Matrix ID | Name | Score | Relative score | Sequence ID | Start | End | Strand | Predicted sequence |
| --- | --- | --- | --- | --- | --- | --- | --- | --- |
| MA0099.1 | MA0099.1.Fos::Jun | 8.071596 | 0.87773 | mm39_ncbiRefSeqCurated_NM_138945.2 | 895 | 902 | + | gtgagtga |
| MA0099.1 | MA0099.1.Fos::Jun | 7.397874 | 0.855421 | mm39_ncbiRefSeqCurated_NM_138945.2 | 705 | 712 | - | atgaataa |
| MA0099.1 | MA0099.1.Fos::Jun | 7.269981 | 0.851186 | mm39_ncbiRefSeqCurated_NM_138945.2 | 900 | 907 | - | **gtcattca** |
| MA0099.1 | MA0099.1.Fos::Jun | 7.124358 | 0.846364 | mm39_ncbiRefSeqCurated_NM_138945.2 | 1568 | 1575 | + | gtgtctca |
| MA0099.1 | MA0099.1.Fos::Jun | 6.96761 | 0.841174 | mm39_ncbiRefSeqCurated_NM_138945.2 | 1895 | 1902 | + | atgagtga |
| MA0099.1 | MA0099.1.Fos::Jun | 6.555591 | 0.827531 | mm39_ncbiRefSeqCurated_NM_138945.2 | 1060 | 1067 | - | atgagaaa |
| MA0099.1 | MA0099.1.Fos::Jun | 6.489908 | 0.825356 | mm39_ncbiRefSeqCurated_NM_138945.2 | 239 | 246 | + | ctgagtct |
| MA0099.1 | MA0099.1.Fos::Jun | 6.489908 | 0.825356 | mm39_ncbiRefSeqCurated_NM_138945.2 | 1494 | 1501 | - | ctgagtct |

atgaataa reverse complement: ttattcat

gtcattca reverse complement: **tgaatgac**

atgagaaa reverse complement: tttctcat

ctgagtct reverse complement: agactcag

MA0099.2.FOS::JUN (excluding overlapping sites)

| Matrix ID | Name | Score | Relative score | Sequence ID | Start | End | Strand | Predicted sequence |
| --- | --- | --- | --- | --- | --- | --- | --- | --- |
| MA0099.2 | MA0099.2.FOS::JUN | 7.665297 | 0.890543 | mm39_ncbiRefSeqCurated_NM_138945.2 | 1311 | 1317 | + | tgaaaca |

### Mutation Strategy:

All 8 binding sites will undergo deletion mutation

### Construction Plan:

Vector: H352 pGL4.10
Wild-type promoter construction: pGL4.10-*Pou4f3* promoter(WT)
Mutant promoter construction: pGL4.10-*Pou4f3* promoter(MUT)
Control: H352 pGL4.10

### Dual Luciferase Assay Groups:

1. pGL4.10 + pcDNA3.1(+)-MCS-3xFLAG + Renilla plasmid co-transfection in HEI-OC1
2. pGL4.10 + pcDNA3.1(+)-*Fos*-3xFLAG + Renilla plasmid co-transfection in HEI-OC1
3. pGL4.10-*Pou4f3* promoter(WT) + pcDNA3.1(+)-MCS-3xFLAG + Renilla plasmid co-transfection in HEI-OC1
4. pGL4.10-*Pou4f3* promoter(WT) + pcDNA3.1(+)-*Fos*-3xFLAG + Renilla plasmid co-transfection in HEI-OC1
5. pGL4.10-*Pou4f3* promoter(MUT) + pcDNA3.1(+)-MCS-3xFLAG + Renilla plasmid co-transfection in HEI-OC1
6. pGL4.10-*Pou4f3* promoter(MUT) + pcDNA3.1(+)-*Fos*-3xFLAG + Renilla plasmid co-transfection in HEI-OC1

## Gfi1

### Promoter 1 Sequence 1: 5'UTR upstream 2000bp + 5'UTR

>mm39_ncbiRefSeqCurated_NM_001267621.1 range=chr5:107873577-107875671 5'pad=0 3'pad=0 strand=- repeatMasking=none

ttattttccctgttaaaaagaatagaagataacaaggtaaagatttgcgttgggactattttgagatagtcacaacaggacacgacaaagcacacaataagtgaattgttcatagaatatgccgttagctaatacagtgtgtgcagcccctctaaaattgcatgcctctgcattcatctataaagaaagggtgtgttgagaatcctgcatctgaggactggttaacagacaggatg*tgtgtcat*agcacatagcagtgatatataggccctatggataaaatgccaaatttattaaaaatcttaaagaaatgctatgctagtcaaatattttgcataaattcagattttttttacagttaaaaatatcactgactttttttccccacagaaagggctttttcttataacgattttgactaatctaaactataatagttgcagggctagttcattgtcaggtgcgtggcgaaagagtgcaaatcccgggggttctttcttcagaatcaacgaggcaatacacttgaacatgtatgtttttgtaatctgcggggcatcacccgtcctccaggatctcatttcataattttggttcacgaaataacgctcaaaatccagcccaactcatttggaagaggactgtgctgacaatggcttcacccctgcgaggagagggtgcacacccacagacacacagcacaatagtggttggattttttatttgcagaagagtgaggtttgagctcctctccccgccctgccatcaatagtgttaatcccacgcctccattgagggcaagaccacggtgggcattattctgaagccttcattttgcaatgactccaaaagtccacatggcctagtaccctgctgcagccccaggcgccacgcctcccaccccaccccaacctggaccaagcaagactcagctgagccgcagctccgaagtcgcctttaatttcccagctccgaccaccggaagcgagcgcgaggcgggggccgggtggggaggacgcgcccaccgatgccgccggcgcccgcgggacgcgccaccagcccggcccttgttcgctgggctcgacgctcgcccacctcttccaaatttaaccgttacctaaatccgaagggaaatgagcaaacctctgggattgggtgtcaaggtattttcagcttcgttgggcgtatttatcctcaagtgtttccacaacaagttatttcggggcccgaggcgcaggtttctttctgcggacgcggtggccagtggccggactccaggccggtggcaccggggtccggtgattcacggccgcgatcggggcgatgctgcccaaacagtcgacaggattggggtgcccacgggagccgtgcgagctgcctgctaccagcgcccgcagctcctgtcaatctgtgtcctgaatctgtgacttctcgctgcggaagtctccaggagccaagaatacttcgattttctttctagtcatttctagtgctgatttctcagacatcccggggactctatagatagccccgcgatcccacagacaaacaaattgggggctgctcaggggtgcccaaggagttggcaaggatacccccttcctgtcacctcctgaccccttgcgcaaaggaggctcgtggcccgctgtcccactgggggatggggctggggttgagaaggctagtgagcgcctctaacgctcaggaagtgaagtttgtggttttgggggctgagctccgaaggagattaaaaaaaaaaaaaaaaaaaaagtcagagagacagatcagtctctcccgaaagcaagattcagtttgcacactctcagagctcggtgcccgcgccctgaccccgaaggtaggtttgctgaactgctgtgcctcccgctcaggcgggggtggcctcgaggactggtaggagtacattgacactgtggggacgtcccgagggcgcaggcgggtccccacctgctgcagagcatcggctgctgggttctggagccacccAAGCGGTGGAGCGAAAGATCTGGAATAGAGGGGCACACAGTTTAGCGGTTAGATAGCAGAGTGGGATCCACGCAGGAAGAAAACATCTGGTGGGC

Construction Synthesis Length: 2095bp

### Promoter 1 Binding Site Prediction Results:

MA0099.1.Fos::Jun (excluding overlapping sites)

| Matrix ID | Name | Score | Relative score | Sequence ID | Start | End | Strand | Predicted sequence |
| --- | --- | --- | --- | --- | --- | --- | --- | --- |
| MA0099.1 | MA0099.1.Fos::Jun | 10.46139 | 0.956861 | mm39_ncbiRefSeqCurated_NM_001267621.1 | 1295 | 1302 | + | gtgattca |
| MA0099.1 | MA0099.1.Fos::Jun | 7.633443 | 0.863221 | mm39_ncbiRefSeqCurated_NM_001267621.1 | 236 | 243 | + | gtgtgtca |
| MA0099.1 | MA0099.1.Fos::Jun | 7.072866 | 0.844659 | mm39_ncbiRefSeqCurated_NM_001267621.1 | 66 | 73 | - | gtgactat |
| MA0099.1 | MA0099.1.Fos::Jun | 6.677878 | 0.831581 | mm39_ncbiRefSeqCurated_NM_001267621.1 | 414 | 421 | + | ttgactaa |
| MA0099.1 | MA0099.1.Fos::Jun | 6.555591 | 0.827531 | mm39_ncbiRefSeqCurated_NM_001267621.1 | 1130 | 1137 | + | atgagcaa |
| MA0099.1 | MA0099.1.Fos::Jun | 6.489908 | 0.825356 | mm39_ncbiRefSeqCurated_NM_001267621.1 | 921 | 928 | - | ctgagtct |
| MA0099.1 | MA0099.1.Fos::Jun | 6.378916 | 0.821681 | mm39_ncbiRefSeqCurated_NM_001267621.1 | 588 | 595 | - | gtgaacca |

gtgactat reverse complement: atagtcac

ctgagtct reverse complement: agactcag

gtgaacca reverse complement: tggttcac

MA0099.2.FOS::JUN (excluding overlapping sites)

| Matrix ID | Name | Score | Relative score | Sequence ID | Start | End | Strand | Predicted sequence |
| --- | --- | --- | --- | --- | --- | --- | --- | --- |
| MA0099.2 | MA0099.2.FOS::JUN | 6.656337 | 0.853715 | mm39_ncbiRefSeqCurated_NM_001267621.1 | 837 | 843 | + | tgactcc |

### Promoter 1 Mutation Strategy:

All 8 binding sites will undergo deletion mutation

### Promoter 1 Construction Plan:

Vector: H352 pGL4.10
Wild-type promoter construction: pGL4.10-*Gfi1* promoter1(WT)
Mutant promoter construction: pGL4.10-*Gfi1* promoter1(MUT)
Control: H352 pGL4.10

### Promoter 1 Dual Luciferase Assay Groups:

1. pGL4.10 + pcDNA3.1(+)-MCS-3xFLAG + Renilla plasmid co-transfection in HEI-OC1
2. pGL4.10 + pcDNA3.1(+)-*Fos*-3xFLAG + Renilla plasmid co-transfection in HEI-OC1
3. pGL4.10-*Gfi1* promoter1(WT) + pcDNA3.1(+)-MCS-3xFLAG + Renilla plasmid co-transfection in HEI-OC1
4. pGL4.10-*Gfi1* promoter1(WT) + pcDNA3.1(+)-*Fos*-3xFLAG + Renilla plasmid co-transfection in HEI-OC1
5. pGL4.10-*Gfi1* promoter1(MUT) + pcDNA3.1(+)-MCS-3xFLAG + Renilla plasmid co-transfection in HEI-OC1
6. pGL4.10-*Gfi1* promoter1(MUT) + pcDNA3.1(+)-*Fos*-3xFLAG + Renilla plasmid co-transfection in HEI-OC1

### Promoter Sequence 2: Alternative promoter (5'UTR upstream 2000bp + 5'UTR)

>mm39_ncbiRefSeqCurated_NM_010278.2 range=chr5:107871705-107874209 5'pad=0 3'pad=0 strand=- repeatMasking=none

gattttctttctagtcatttctagtgctgatttctcagacatcccggggactctatagatagccccgcgatcccacagacaaacaaattgggggctgctcaggggtgcccaaggagttggcaaggatacccccttcctgtcacctcctgaccccttgcgcaaaggaggctcgtggcccgctgtcccactgggggatggggctggggttgagaaggctagtgagcgcctctaacgctcaggaagtgaagtttgtggttttgggggctgagctccgaaggagattaaaaaaaaaaaaaaaaaaaaagtcagagagacagatcagtctctcccgaaagcaagattcagtttgcacactctcagagctcggtgcccgcgccctgaccccgaaggtaggtttgctgaactgctgtgcctcccgctcaggcgggggtggcctcgaggactggtaggagtacattgacactgtggggacgtcccgagggcgcaggcgggtccccacctgctgcagagcatcggctgctgggttctggagccacccaagcggtggagcgaaagatctggaatagaggggcacacagtttagcggttagatagcagagtgggatccacgcaggaagaaaacatctggtgggcatgtggcggcagcgggcttctggaggcctccttctgagcgccttgccttcagctcccgaatttccagcctcagatgaccaggggaagcctggcgggccgctgtgccaggagcgtgccgccccgccccctggacgccagctgcagcacgaggttgccttcccacgcgctgggcctccttgccatccccttacccctcctacccggaacccagcaaagccctccgaagcgtaggtttgtggcctgggcgaggacgccacctctgtgtctaccctgtcccgcgtggtgctgacgcgcagacccagcgcgggaccagacagccctgcttcccacgggtcccgttcttggctatcgcaggaccgaacctcatcgaggcctccagaactcttgccctgttttcttgctcttcatgcttgctttaagaattgtcctctggttggagcgtctggctgtacagacagggagtgcgggaaaattaaagcgccggagcctgggttctgggagccaaacccgaccaaggcgtgtgactatacattatgactgggaatctgaagcaggagtccttgggctccttttaagtgggtacaacagggtgtgttctgtacgcgctcctgggggggggtggcgaagaggctcgggttcctcagtctgaactgcacgaagcccaaagacccggggcgtgcgccggcctgtgccccagtctttgagcccgccgccgccgttcagcctctattgccttggaccttggaggcaggcggattcgtttcaccctaggcctccctagggtcgcccccggagcgggccaggagctgggacgcagtctcagccccgcctcctgcagggctcaggtttcgacccgagattaggtgaactgattgggggttaaagagagcgacgccagctctcggtcatgctcagggaccctggcctcctggccaggccttctggccactgctcactcgtgcgcgctccggtttcatcttgctggcccgcgaagtcgttggcactcattaaatttgggcaccgcgtgtttgccagtaagcacgcagtttaaactcaacttccctaggacaagatttttggtgagcagatcagaaaataattctttctgggacttagtttctgaagcaaggcctacaggtctggaatgtttctcagtggtcgccgtcctggccggtgccacaccactattccagtgggcgtttctcctgggacaagtgttggtcctttgccactcccacctcactttcctgtgcctctggcttcctgacttctgctccagccactgcagcacatagtccctaggcaaaccagtccctagtacgcccctctttcgcttctgttcgtcgcggggctcctggaggttttggCTCCAAGCCCCTTCCGGGTCCCAAACACTGATGCCCCCTGACTGGCTAAACTAAGCCACGCATCCCTGGCG**TGAGCCAC**AACCCAGAAGGGAGCAGGTGGGCTTGCAGGGTTGACTTGGGATAACGGACCAGTTGTGGACTACTGCTCTCAGGAGAGTGATGATCTAGCTTTGGTAGGGAAGGGGAGGGGCTGAGGCGTGGGCAGGGCAGAGCAAAGGGACCAGAGCCAGAGCCTGGGGACAGGTTTTACCACTGAGCTGTTGCAGTGGCGGCGGAGGCCGGGATTCGTGCCACCTGTCCGAGTGCCACCTGGTGAGCGTGGCGCCTGGGTCCAGGCCCCTCCTCCCGCGGCTTCCCTCTCTCCTCCCTGGCCCACACTCTTCCTTGGCCTGGGAACCTACCACAACCGCCATCGGTGCTGACCCTCGTTTCCACCCAATTTTCCCCCTTCTCTCAGAACTCAGAGTATCCGAGGGTCCAAACATTCGTCCAGCGGCTGACCACC

Construction Synthesis Length: 2505bp

### Promoter 2 Binding Site Prediction Results:

MA0099.1.Fos::Jun (excluding overlapping sites)

| Matrix ID | Name | Score | Relative score | Sequence ID | Start | End | Strand | Predicted sequence |
| --- | --- | --- | --- | --- | --- | --- | --- | --- |
| MA0099.1 | MA0099.1.Fos::Jun | 8.650418 | 0.896896 | mm39_ncbiRefSeqCurated_NM_010278.2 | 2071 | 2078 | + | GTGAGCCA |
| MA0099.1 | MA0099.1.Fos::Jun | 7.072866 | 0.844659 | mm39_ncbiRefSeqCurated_NM_010278.2 | 1154 | 1161 | + | gtgactat |

MA0099.2.FOS::JUN (excluding overlapping sites)

| Matrix ID | Name | Score | Relative score | Sequence ID | Start | End | Strand | Predicted sequence |
| --- | --- | --- | --- | --- | --- | --- | --- | --- |
| MA0099.2 | MA0099.2.FOS::JUN | 6.686689 | 0.854823 | mm39_ncbiRefSeqCurated_NM_010278.2 | 1180 | 1186 | + | tgaagca |
| MA0099.2 | MA0099.2.FOS::JUN | 6.686689 | 0.854823 | mm39_ncbiRefSeqCurated_NM_010278.2 | 1754 | 1760 | + | tgaagca |

### Promoter 2 Mutation Strategy:

All 5 binding sites will undergo deletion mutation

### Promoter 2 Construction Plan:

Vector: H352 pGL4.10

Wild-type promoter construction: pGL4.10-*Gfi1* promoter2(WT)

Mutant promoter construction: pGL4.10-*Gfi1* promoter2(MUT)

Control: H352 pGL4.10

### Promoter 2 Dual Luciferase Assay Groups:

1. pGL4.10 + pcDNA3.1(+)-MCS-3xFLAG + Renilla plasmid co-transfection in HEI-OC1
2. pGL4.10 + pcDNA3.1(+)-*Fos*-3xFLAG + Renilla plasmid co-transfection in HEI-OC1
3. pGL4.10-*Gfi1* promoter2(WT) + pcDNA3.1(+)-MCS-3xFLAG + Renilla plasmid co-transfection in HEI-OC1
4. pGL4.10-*Gfi1* promoter2(WT) + pcDNA3.1(+)-*Fos*-3xFLAG + Renilla plasmid co-transfection in HEI-OC1
5. pGL4.10-*Gfi1* promoter2(MUT) + pcDNA3.1(+)-MCS-3xFLAG + Renilla plasmid co-transfection in HEI-OC1
6. pGL4.10-*Gfi1* promoter2(MUT) + pcDNA3.1(+)-*Fos*-3xFLAG + Renilla plasmid co-transfection in HEI-OC1

# Enhancer Gene

## Atoh1 enhancer 1

### *Atoh1* enhancer 1 Sequence:

TCCAAGGTCCGGCAATGAAGTTTGCATAACAAACGTTTGGCAGCTCCCTCTCTCACACCCCATTAACAAGCTGTAACATATAGCTGCAGGTTGCTATAATCTCATTAATATTTTGGAAACTTGAATATTGAGTATTTCTGAGCGCTCATTCCCCATATGCCAGACCACTCCTGCCATGCTGACTGGTTCCTTTCTCTCCATTATTAGCAATTAGCTTCTACCTTCCAAAGTCAGATCCAAGTATCTAAGATACTACCAAAGGCATCAACTATGTATGCAAGTTAGGCATGCTTAATATCACCCAAACAAACAAAGAGTCAGCACTTCTTAAAGTAATGAAGATAGATAAATCGGGTTAGTTCTTTGGGACACCGCTGTTGTTTTCCAGAGTTTTTCTATACTTTAAGCAGCTTGTTTTAT

ATTCTGTCTTTGCCCTCAGCCAGCTAACATTTTATTTGTTGAGGGTTTTGGCTCACCACACTTTTGGAAACTTATTTGATTTCACGGGGAGCTGAAGGAAGATTGTTTTTGGCAACAGGCAAGTTTAACACGTTCTTCATGGGGCATTGCGAATGGCACATCTACCAGAAAGGGAGGGGGAGTAACTTCCTCGTGCTGAACCAGCAGGAGACCAGAGCTTTCCTGAGGTCTTCCTATTGATTTTAAAGATTTAAAACTGAGCCCCAAAGTTGTAATGTTATTGAAGTTTGTCTTGGAATATACATCTCCTCTGCTAACTTAAAAGTTCAAGAAAGGAAAGGAAAGAAATAGAACCCCTTGCTAACTACAACCTAGACTGAGAGGTGAAGATCGCGGGCAAAGACAGGTGGTCACTGAAACGTTTGCAGTTCTTTTCTTCCGAAGGCTTAGGACACAGGGTAAGGAGGAGCTAAAATAAAGCCGAGTGTACGTTTAGTCTTCTCTGCACCCCAGGCCTAGTGTCTCCCCAGGCAAGGAGTCACCCCCTTTGCTTCTGGCTCCTAACTGAAAAAGGCAAAAGGGAGTGGAGAATGGGTTAAATCCCAGGACACAGGGGAGAGGCAGGGGAGGAGAGAAGTCGGAGGAAGATAAAGGAAAGGACAGGAACCAAGAAGCGTGGGGGTAGTTTGCCGTAATGTGAGTGTTTCTTAATTAGAGAGCGGCTGACAATAGAGGGGCTGGCAGAGGCTCCTGGCCCCGGTGCGGAGCGT

CTGGAGCGGAGCACGCGCTGTCAGCTGGTGAGCGCACTCGCTTTCAGGCCGCTCCCCGGGGAGCTGAGCGGCCACATTTAACACCGTCGTCACCCTCCCCGGCCTCCTCAACATCGGCCTCCTCCTCGTAGACAGCCTTGCTCGGCCCCCCACCGGCAGAGTTTACAGAAGCCAGAGCCTCTCGCCGTTCCCCCGCATTCGCCCGGGCCCCTCTGGTCCCCAGCTGCGCAGCGGGAGCCGCCACTGCCCACTGCACCTCCCGGCAACTAACAGCCCCGCCAGAAAAGGAGCATGCTTAAGCCAGAGTCAAGCAAAGCCTAGGGGAGG

### Enhancer 1 Binding Site Prediction Results:

MA0099.1.Fos::Jun (excluding overlapping sites)

| Matrix ID | Name | Score | Relative score | Sequence ID | Start | End | Strand | Predicted sequence |
| --- | --- | --- | --- | --- | --- | --- | --- | --- |
| MA0099.1 | MA0099.1.Fos::Jun | 5.968879 | 0.808104 | AF218258.1 | 282 | 289 | - | ATGCCTAA |
| MA0099.1 | MA0099.1.Fos::Jun | 6.4557962 | 0.824226 | AF218258.1 | 313 | 320 | + | AAGAGTCA |
| MA0099.1 | MA0099.1.Fos::Jun | 8.650418 | 0.896895 | AF218258.1 | 469 | 476 | - | GTGAGCCA |
| MA0099.1 | MA0099.1.Fos::Jun | 6.56894 | 0.827973 | AF218258.1 | 598 | 605 | + | GGGAGTAA |
| MA0099.1 | MA0099.1.Fos::Jun | 7.124358 | 0.846364 | AF218258.1 | 955 | 962 | - | GTGACTCC |

ATGCCTAA reverse complement: TTAGGCAT

GTGAGCCA reverse complement: GTGAGCCA

GTGACTCC reverse complement: GTGACTCC

MA0099.2.FOS::JUN (excluding overlapping sites)

| Matrix ID | Name | Score | Relative score | Sequence ID | Start | End | Strand | Predicted sequence |
| --- | --- | --- | --- | --- | --- | --- | --- | --- |
| MA0099.2 | MA0099.2.FOS::JUN | 5.588805 | 0.814747 | AF218258.1 | 102 | 108 | - | TTAATGA |
| MA0099.2 | MA0099.2.FOS::JUN | 5.6307364 | 0.816278 | AF218258.1 | 180 | 186 | + | TGACTGG |
| MA0099.2 | MA0099.2.FOS::JUN | 5.7285447 | 0.819848 | AF218258.1 | 617 | 623 | + | TGAACCA |
| MA0099.2 | MA0099.2.FOS::JUN | 7.6144814 | 0.888688 | AF218258.1 | 1493 | 1499 | - | TGACTCT |

TTAATGA reverse complement: TCATTAA

TGACTCT reverse complement: AGAGTCA

### Enhancer 1 Mutation Strategy:

All 14 binding sites will undergo deletion mutation

### Enhancer 1 Construction Plan:

Vector: H352 pGL4.10

Wild-type promoter construction: pGL4.10-*Atoh1* enhancer1(WT)

Mutant promoter construction: pGL4.10- *Atoh1* enhancer1(MUT)

Control: H352 pGL4.10

### Enhancer 1 Dual Luciferase Assay Groups:

1. pGL4.10 + pcDNA3.1(+)-MCS-3xFLAG + Renilla plasmid co-transfection in HEI-OC1
2. pGL4.10 + pcDNA3.1(+)-*Fos*-3xFLAG + Renilla plasmid co-transfection in HEI-OC1
3. pGL4.10- *Atoh1* enhancer1(WT) + pcDNA3.1(+)-MCS-3xFLAG + Renilla plasmid co-transfection in HEI-OC1
4. pGL4.10- *Atoh1* enhancer1(WT) + pcDNA3.1(+)-*Fos*-3xFLAG + Renilla plasmid co-transfection in HEI-OC1
5. pGL4.10- *Atoh1* enhancer1(MUT) + pcDNA3.1(+)-MCS-3xFLAG + Renilla plasmid co-transfection in HEI-OC1
6. pGL4.10- *Atoh1* enhancer1(MUT) + pcDNA3.1(+)-*Fos*-3xFLAG + Renilla plasmid co-transfection in HEI-OC1

## Atoh1 enhancer 2

### Enhancer 2 Sequence:

AGGCAGCTCCATTCTTGGGGCGTGCATAAAATACTTCCCACTCCTTCTCAGGGGTTAAAACCCATCTCCTTTCAAACTTGACTCTAAGTTCTCCGGGGCGCTCAGTGACTCAGCTGTCTGATCATCCCTTCCTCTGTTTTTAACCAAGTCTTTCTCTTTTACTCATTCTATGCAACCTGAATAGTTTGGAATGCCAGCCCAGAGAGTGCGCCTTTCTTCCTACTGCGACCAGAAAGGCAGCGGGGATCATGTGACGCGCATTACTCTCGCCGACTTGTTAATATCAGCTGTGAATTTTTCATGACGGCTTCGGTAACCTGTAGCCTGGAGTTGAAGCTCTGGGGGTTTTCACCACAAAGAGGTGGATCAGCTGAGCTCTCACTTCCCATCTGGCACTGGGTGACAGGGACAGTGAGGGGGGCGGCAGAAAACCTGGGGTTGTGGGGCAGCCATTGTCGAGCATCAAAGGCTAAACATGTGCTGTTGTGTGCTCGTATCTTTGAGACACTTGTTTATCACTGGCTTCAGAATACACGGAGCCAGCGTGTTTAACTTTATAGTCACCAGAACAGCTGCCGCGCGTGTAAGTAATACCCTTGCACACAGTGGCATCCTGATCTGCCAAGCCAGGCTCAGGACCTCTCTGAGTGGCCGCTTTTGTGCTATCTGGCATGCTCCTTTACCCACGCAGGCAGGCATCCACGTGCAGGCACACACACAGACGCACACCCTAGGCATGCAGGGCTGTTTGAAACTGAAAACCCTCCAAGCTAATTCCCATTTGTAACGGAATTAAGCTGGATTTTATGTTTTAAACTTTAAAAAGAGAGAGAGAGAGAGAGAATGTTTTGAAAGTGAGAGTCTCAAACGTATTCAAAGTCAAAGCGGGAGGAGAGTGGGGAGAAAAAAGCTAGTTAAAAAAAAAAGATAAAATTTGAGTAGTTTTGTAGAATCACAACACACCACACCTGAATGAGGAAAAAGAAAGAAAAGGGGAAATAACAGCGGTGCTACAATGCACCTGTGC

### Enhancer 1 Binding Site Prediction Results:

MA0099.1.Fos::Jun (excluding overlapping sites)

| Matrix ID | Name | Score | Relative score | Sequence ID | Start | End | Strand | Predicted sequence |
| --- | --- | --- | --- | --- | --- | --- | --- | --- |
| MA0099.1 | MA0099.1.Fos::Jun | 11.255118 | 0.983143 | *Atoh1* eh2 | 105 | 112 | + | GTGACTCA |
| MA0099.1 | MA0099.1.Fos::Jun | 9.669376 | 0.930635 | *Atoh1* eh2 | 159 | 166 | - | ATGAGTAA |
| MA0099.1 | MA0099.1.Fos::Jun | 6.56894 | 0.827973 | *Atoh1* eh2 | 261 | 268 | - | GAGAGTAA |
| MA0099.1 | MA0099.1.Fos::Jun | 7.124358 | 0.846364 | *Atoh1* eh2 | 501 | 508 | - | GTGTCTCA |
| MA0099.1 | MA0099.1.Fos::Jun | 6.3567653 | 0.820947 | *Atoh1* eh2 | 512 | 519 | - | GTGATAAA |
| MA0099.1 | MA0099.1.Fos::Jun | 7.0728655 | 0.844659 | *Atoh1* eh2 | 557 | 564 | - | GTGACTAT |
| MA0099.1 | MA0099.1.Fos::Jun | 6.6426015 | 0.830412 | *Atoh1* eh2 | 584 | 591 | + | GTAAGTAA |
| MA0099.1 | MA0099.1.Fos::Jun | 6.6426015 | 0.830412 | *Atoh1* eh2 | 680 | 687 | - | GTGGGTAA |
| MA0099.1 | MA0099.1.Fos::Jun | 7.2699814 | 0.851186 | *Atoh1* eh2 | 949 | 956 | - | GTGATTCT |

ATGAGTAA reverse complement: TTACTCAT

GAGAGTAA reverse complement: TTACTCTC

GTGTCTCA reverse complement: GTGTCTCA

GTGATAAA reverse complement: TTTATCAC

GTGACTAT reverse complement: ATAGTCAC

TTACCCAC reverse complement: GTGGGTAA

GTGATTCT reverse complement: AGAATCAC

MA0099.2.FOS::JUN (excluding overlapping sites)

| Matrix ID | Name | Score | Relative score | Sequence ID | Start | End | Strand | Predicted sequence |
| --- | --- | --- | --- | --- | --- | --- | --- | --- |
| MA0099.2 | MA0099.2.FOS::JUN | 7.6144814 | 0.888688 | *Atoh1* eh2 | 79 | 85 | + | TGACTCT |
| MA0099.2 | MA0099.2.FOS::JUN | 5.548656 | 0.813282 | *Atoh1* eh2 | 363 | 369 | + | TGGATCA |
| MA0099.2 | MA0099.2.FOS::JUN | 5.6815515 | 0.818133 | *Atoh1* eh2 | 546 | 552 | - | TTAAACA |
| MA0099.2 | MA0099.2.FOS::JUN | 5.548559 | 0.813278 | *Atoh1* eh2 | 936 | 942 | - | CTACTCA |
| MA0099.2 | MA0099.2.FOS::JUN | 7.5725503 | 0.887157 | *Atoh1* eh2 | 970 | 976 | + | TGAATGA |

TTAAACA reverse complement: TGTTTAA

CTACTCA reverse complement: CTACTCA

### Enhancer 2 Mutation Strategy:

All 14 binding sites will undergo deletion mutation

### Enhancer 2 Construction Plan:

Vector: H352 pGL4.10

Wild-type promoter construction: pGL4.10-*Atoh1* enhancer2(WT)

Mutant promoter construction: pGL4.10- *Atoh1* enhancer2(MUT)

Control: H352 pGL4.10

### Enhancer 2 Dual Luciferase Assay Groups:

1. pGL4.10 + pcDNA3.1(+)-MCS-3xFLAG + Renilla plasmid co-transfection in HEI-OC1
2. pGL4.10 + pcDNA3.1(+)-*Fos*-3xFLAG + Renilla plasmid co-transfection in HEI-OC1
3. pGL4.10- *Atoh1* enhancer2(WT) + pcDNA3.1(+)-MCS-3xFLAG + Renilla plasmid co-transfection in HEI-OC1
4. pGL4.10- *Atoh1* enhancer2(WT) + pcDNA3.1(+)-*Fos*-3xFLAG + Renilla plasmid co-transfection in HEI-OC1
5. pGL4.10- *Atoh1* enhancer2(MUT) + pcDNA3.1(+)-MCS-3xFLAG + Renilla plasmid co-transfection in HEI-OC1
6. pGL4.10- *Atoh1* enhancer2(MUT) + pcDNA3.1(+)-*Fos*-3xFLAG + Renilla plasmid co-transfection in HEI-OC1

## Atoh1 enhancer 3

### Enhancer 3 Sequence:

TGGTTGTTCATATGCCATATGTTAAGGAGTTCCCCGAGTGGAACATGTCTAAAGAATGATATGTCTTACTATAACCTCCCTGAAATTTTCACTTAAAAAGAAACAAATCAAAAGACATCTCATGCTTGAATTAAAGGTCAATGAAACAGTGAAGGCCTATGGGTCAGGCACAAAGAAGCCACAGAAGAGTCAAACGCCACTGGTCTCGGAGCCTAGCTTGCCACACATAACCATGGTTAGCATGCTCCTTCTCCACAGTTGGTAGCAATCAGATACATATTTAATGTGGTTTCATTATATCCAATAAATGCTGGCAATAGAATAACCTCCCCAGGGTATTCGTGGAAGAATAGAACAGAGAAAACGATGAGCACATTTAGCACACGCACCCACAGCCTCCTGATAAAAGCCAGCACTTCCCCCATACAGCACCTGTTGTCCTAGCTAGTTTAATTACTTTTCATTGTAGTCAGCAGCCTGAAATATTTATCATGGATCCTATAATTTTCCTAATCCAAAACTGACATCTACTCCAGTGACCACAGGGACTGAATTTTTACCAACTCCTCGTCCCCCTCTTCACCCGCCCCGTGCTTCATTCTCAAGATTAAAGTGAGGAAATTGTTTTTAAGAGAAGCAGCCTTCAGTCAAGTGCCGGCTGCATTGGCAGATGCTAGAATGCTCACACACCAGCCAGCCAGCCAGCCAGCTCTGACAAAGGACAGAAATTAATTTCACTGTGGAAGTGTAAAACATTCAAT

### Enhancer 3 Binding Site Prediction Results:

MA0099.1.Fos::Jun (excluding overlapping sites)

| Matrix ID | Name | Score | Relative score | Sequence ID | Start | End | Strand | Predicted sequence |
| --- | --- | --- | --- | --- | --- | --- | --- | --- |
| MA0099.1 | MA0099.1.Fos::Jun | 6.5294576 | 0.826666 | *Atoh1* eh3 | 159 | 166 | + | ATGGGTCA |
| MA0099.1 | MA0099.1.Fos::Jun | 6.4557962 | 0.824226 | *Atoh1* eh3 | 185 | 192 | + | AAGAGTCA |
| MA0099.1 | MA0099.1.Fos::Jun | 7.2699814 | 0.851186 | *Atoh1* eh3 | 591 | 598 | + | GTGCTTCA |
| MA0099.1 | MA0099.1.Fos::Jun | 6.7202272 | 0.832982 | *Atoh1* eh3 | 613 | 620 | + | GTGAGGAA |

MA0099.2.FOS::JUN (excluding overlapping sites)

| Matrix ID | Name | Score | Relative score | Sequence ID | Start | End | Strand | Predicted sequence |
| --- | --- | --- | --- | --- | --- | --- | --- | --- |
| MA0099.2 | MA0099.2.FOS::JUN | 5.6357985 | 0.816463 | *Atoh1* eh3 | 127 | 133 | + | TGAATTA |
| MA0099.2 | MA0099.2.FOS::JUN | 7.6652966 | 0.890543 | *Atoh1* eh3 | 142 | 148 | + | TGAAACA |
| MA0099.2 | MA0099.2.FOS::JUN | 6.2942185 | 0.840496 | *Atoh1* eh3 | 219 | 225 | + | TGCCACA |
| MA0099.2 | MA0099.2.FOS::JUN | 8.680231 | 0.927590 | *Atoh1* eh3 | 644 | 650 | - | TGACTGA |

TGACTGA reverse complement: TCAGTCA

### Enhancer 3 Mutation Strategy:

All 8 binding sites will undergo deletion mutation

### Enhancer 3 Construction Plan:

Vector: H352 pGL4.10

Wild-type promoter construction: pGL4.10-*Atoh1* enhancer3(WT)

Mutant promoter construction: pGL4.10- *Atoh1* enhancer3(MUT)

Control: H352 pGL4.10

### Enhancer 3 Dual Luciferase Assay Groups:

1. pGL4.10 + pcDNA3.1(+)-MCS-3xFLAG + Renilla plasmid co-transfection in HEI-OC1
2. pGL4.10 + pcDNA3.1(+)-*Fos*-3xFLAG + Renilla plasmid co-transfection in HEI-OC1
3. pGL4.10- *Atoh1* enhancer3(WT) + pcDNA3.1(+)-MCS-3xFLAG + Renilla plasmid co-transfection in HEI-OC1
4. pGL4.10- *Atoh1* enhancer3(WT) + pcDNA3.1(+)-*Fos*-3xFLAG + Renilla plasmid co-transfection in HEI-OC1
5. pGL4.10- *Atoh1* enhancer3(MUT) + pcDNA3.1(+)-MCS-3xFLAG + Renilla plasmid co-transfection in HEI-OC1
6. pGL4.10- *Atoh1* enhancer3(MUT) + pcDNA3.1(+)-*Fos*-3xFLAG + Renilla plasmid co-transfection in HEI-OC1

**Supplemental Table 1. Small Molecules**

| **Reagent** | **Concentration** | **Vendor** |
| --- | --- | --- |
| Laduviglusib (CHIR-99021) | 3 μM | Med Chem Express (Cat#HY-10182) |
| IWP-2 | 5 μM | Med Chem Express (Cat#HY-13912) |
| T-5224 | 10 μM | Med Chem Express (Cat#HY-12270) |
| EdU (5-ethynyl-2’-deoxyuridine) | 10 μM | Life Technologies (Cat# C10340) |
| 4-OHT (4-Hydroxytamoxifen) | 5 nM | Med Chem Express (Cat# HY-16950B) |
| IDPN(3,3'-Iminodipropionitrile) | 5 μl/g | TCI Shanghai (Cat#TCI-I0010) |
| Tamoxifen | 0.20 mg/g body weight | Sigma-Aldrich (Cat# T5648) |
| Aphidicolin | 1 μM | Med Chem Express (Cat# HY-N6733) |

**Supplemental Table 2. Antibodies**

| **Antibody** | **Source** | **Cat; RRID; Application** | **Dilution** |
| --- | --- | --- | --- |
| DAPI | Thermo Fisher Scientific | Cat# D1306; AB_2629482; IF | 1:800 |
| Phalloidin | Thermo Fisher Scientific | Cat# A22287; AB_2620155; IF | 1:200 |
| Rabbit anti-Myo7a | Proteus Biosciences | Cat# 25-6790; AB_10015251; IF/WB | 1:800 |
| Goat anti-Ocm | Santa Cruz Biotechnology | Cat# sc-7446; AB_2267583; IF/WB | 1:100 |
| Mouse IgG1 anti-Parvalbumin | Sigma-Aldrich | Cat# P3088; AB_477329; IF | 1:200 |
| Mouse IgG2A anti-Tuj1/beta Tubulin 3 | Neuromics | Cat# MO15013; AB_2737114; IF/WB | 1:200 |
| Mouse IgG1 anti-CtBP2 | BD Biosciences | Cat# 612044; AB_399431; IF/WB | 1:200 |
| Goat anti-Sox2 | Santa Cruz Biotechnology | Cat# sc-17320; AB_2286684; IF/WB | 1:200 |
| Rat anti-Sox2 | Thermo Fisher Scientific | Cat# 14-9811-82; AB_11219471; IF/WB | 1:200 |
| Mouse anti-Green Fluorescent Protein (GFP) | Sigma-Aldrich | Cat# G6539; AB_259941; IF/ELISA | 1:200 |
| Mouse IgG1 (κ light chain) anti-Jagged1 | Santa Cruz Biotechnology | Cat# sc-390177; AB_2892141; IF/WB | 1:200 |
| Mouse anti-Ki-67 | Abcam | Cat# ab279653; AB_2934265; IF/WB | 1:200 |
| Rabbit anti-c-Fos | Cell Signaling Technology | Cat# 2250; AB_2247211; IF/WB/ChIP | 1:100 (IF)  1:50 (CUT&Tag) |
| Rat IgG2a (κ light chain) anti-c-Fos | Synaptic Systems | Cat# 226 017; AB_2864765 | 1:200 (IF) |
| Alexa Fluor™ Donkey anti-Goat IgG 488 | Thermo Fisher Scientific | Cat# A11055; AB_2534102 | 1:500 |
| Alexa Fluor™ Donkey anti-Goat IgG 555 | Thermo Fisher Scientific | Cat# A21432; AB_2535853 | 1:500 |
| Alexa Fluor™ Donkey anti-Goat IgG 647 | Thermo Fisher Scientific | Cat# A21447; AB_2535864 | 1:500 |
| Alexa Fluor™ Donkey anti-Mouse IgG 488 | Thermo Fisher Scientific | Cat# A21202; AB_141607 | 1:500 |
| Alexa Fluor™ Donkey anti-Mouse IgG 555 | Thermo Fisher Scientific | Cat# A31570; AB_2536180 | 1:500 |
| Alexa Fluor™ Donkey anti-Mouse IgG 647 | Thermo Fisher Scientific | Cat# A31571; AB_162542 | 1:500 |
| Alexa Fluor™ Donkey anti- Rabbit IgG 405 | Thermo Fisher Scientific | Cat# A48258; AB_2890547 | 1:500 |
| Alexa Fluor™ Donkey anti-Rabbit IgG 488 | Thermo Fisher Scientific | Cat# A21206; AB_2535792 | 1:500 |
| Alexa Fluor™ Donkey anti-Rabbit IgG 555 | Thermo Fisher Scientific | Cat# A31572; AB_162543 | 1:500 |
| Alexa Fluor™ Donkey anti-Rabbit IgG 647 | Thermo Fisher Scientific | Cat# A31573; AB_2536183 | 1:500 |
| Alexa Fluor™ Donkey anti-Rat IgG 488 | Thermo Fisher Scientific | Cat# A21208; AB_2535794 | 1:500 |
| Alexa Fluor™ Donkey anti-Rat IgG 555 | Thermo Fisher Scientific | Cat# A48270; AB_2896336 | 1:500 |

**Supplemental Table 3. Primers**

| **Gene** | **Forward primer** | **Reverse primer** |
| --- | --- | --- |
| *Atoh1* | GGGGTTGTAGTGGACGAGC | CGTTGTTGAAGGACGGGATAAC |
| *Gfi1* | AGAAGGCGCACAGCTATCAC | GGCTCCATTTTCGACTCGC |
| *Pou4f3* | CCCAAATTCTCCAGCCTACA | CCAGCAGGCTCTCATCAAA |
| *Fos* | TACTACCATTCCCCAGCCGA | GCTGTCACCGTGGGGATAAA |
| *Jag1* | TGTGCAAACATCACTTTCACCTTT | GCAAATGTGTTCGGGTGGTAAGAC |


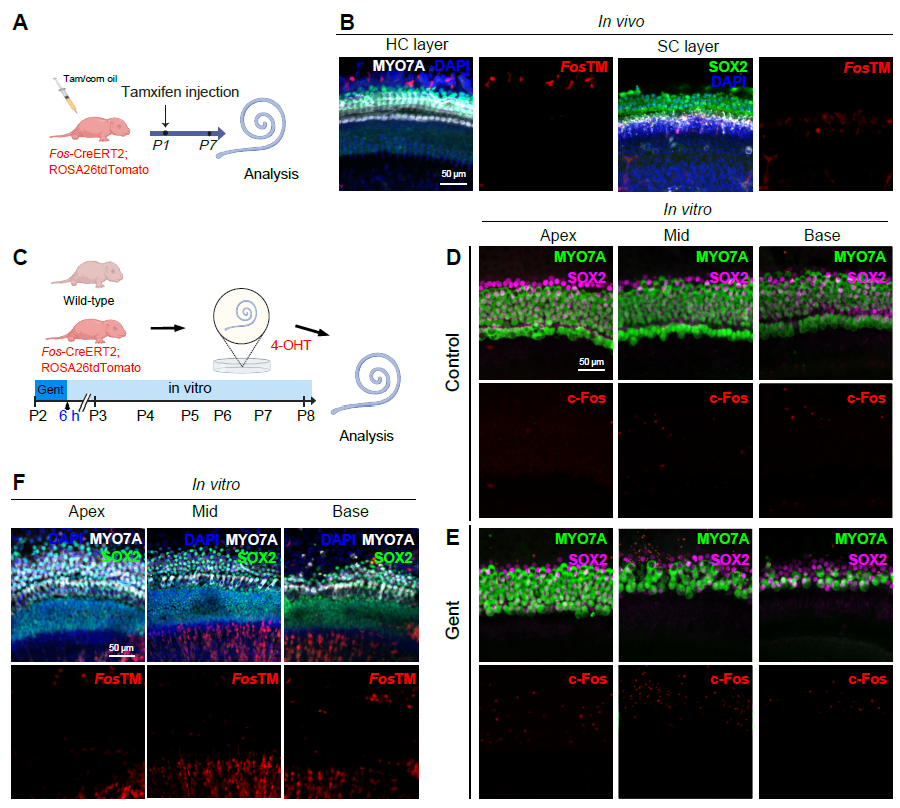
**Supplementary figures and legends.**

**Figure S1: Limited *Fos* activation in the cochlea during development and after injury.**

(A-B) In vivo lineage tracing in the developing cochlea. (A) Schematic: *Fos*TM mice received tamoxifen at P1, and cochleae were collected at P7. (B) Representative images show *Fos*-lineage labeling (*Fos*TM, red) is absent from the sensory epithelium containing hair cells (MYO7A, white) and supporting cells (SOX2, green).

(C-F) In vitro analysis of Fos activation in injured cochleae. (C) The schematic details the design for two parallel experiments using P2 cochleae. For the c-Fos protein analysis, wild-type explants were used; for lineage tracing, *Fos*TM explants were used. Both were cultured for 5 days after a 6h gentamicin treatment. (D, E) In wild-type explants, direct immunofluorescence for c-Fos protein (red) shows no induction when comparing the (D) control (undamaged) to the (E) gentamicin-damaged condition. (F) In the parallel experiment with *Fos*TM explants, representative images confirm that *Fos*-lineage labeling remains sparse and does not increase after injury.

Scale bars = 50 μm.


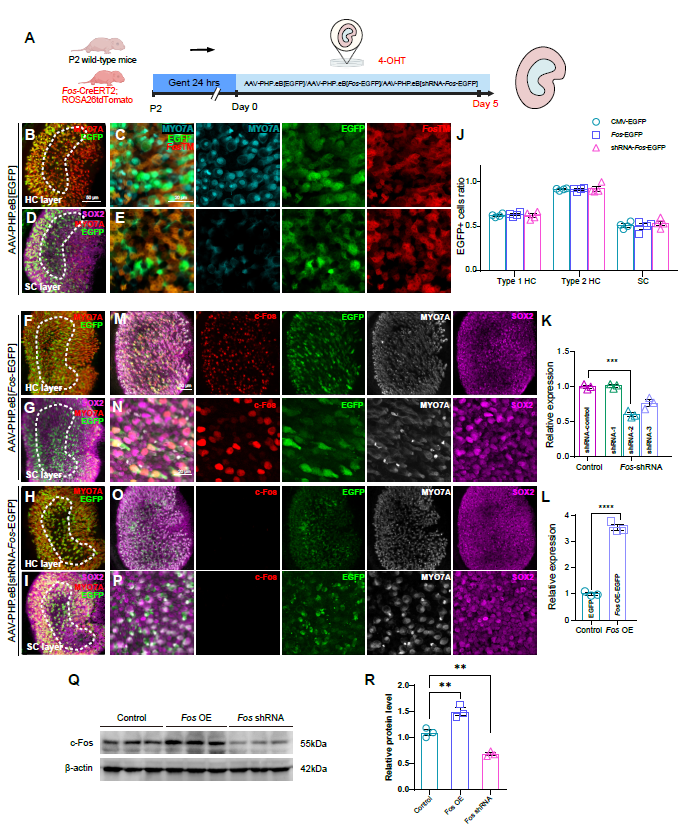


**Figure S2: Validation of AAV-PHP.eB-mediated transduction and gene regulation in utricular explants.**

(A) Experimental workflow schematic. P2 utricles were damaged with gentamicin, then transduced with one of three AAV constructs (AAV-CMV-EGFP, AAV-*Fos* OE, or AAV-*Fos* shRNA). 4-OHT was present throughout the culture period to facilitate lineage tracing in *Fos*TM reporter explants.

(B-I) Representative confocal images demonstrating AAV transduction efficiency in the hair cell layers and supporting cell layers of the utricular sensory epithelium. All images show EGFP expression (green) indicating transduced cells, co-labeled with MYO7A (hair cells, cyan) and *Fos*-lineage tracing (red)*.* (C, E) High-magnification of the utricle striolar region.

(J) Quantification of transduction efficiency. Bar graph showing the percentage of transduced (EGFP+) cells within Type I hair cells, Type II hair cells, and supporting cells for each AAV construct. (two-way ANOVA using Tukey's multiple comparisons test; n = 4 per condition).

(K-L) qPCR validation of AAV-mediated *Fos* gene regulation. (K) The selected AAV-Fos shRNA construct significantly downregulated *Fos* mRNA levels compared to controls (one-way ANOVA with Tukey’s post-hoc test, n = 3 per condition). (L) The AAV-*Fos* OE construct significantly upregulated *Fos* mRNA levels (unpaired Student's t-test, n = 3 per condition).

***p* < 0.01, ****p* < 0.001, *****p* < 0.0001. Data are shown as the mean ± SEM.

(M-P) Immunofluorescence validation of c-Fos protein regulation. Representative images show that c-Fos protein staining (e.g., white) is strongly activated by AAV-*Fos* OE (M, with high-magnification of the striolar region in N) and decreased by AAV-*Fos* shRNA (O, with high-magnification of the striolar region in P).

(Q-R) Western blot validation of c-Fos protein regulation. (Q) A representative blot and (R) corresponding quantification confirm that c-Fos protein levels significantly increased with AAV-*Fos* OE and decreased with AAV-*Fos* shRNA, relative to the EGFP control (one-way ANOVA with Tukey’s post-hoc test; n = 3 per group).

Scale bars = 50 μm (low magnification) and 20 μm (high magnification).


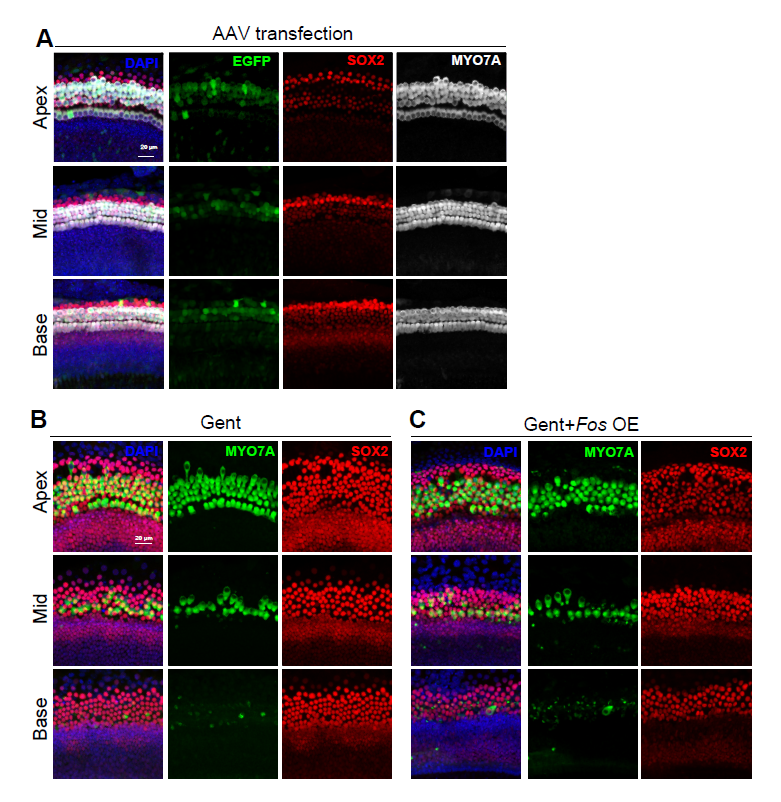


**Figure S3: *Fos* overexpression fails to promote hair cell regeneration in cochlear explants.**

(A) Representative image shows robust transduction efficiency in the cochlea, indicated by EGFP expression (green).

(B-C) Representative confocal images of cultured cochlear explants from the (B) AAV-CMV-EGFP control group and (C) the AAV-*Fos* OE group. Tissues are co-labeled for hair cells (MYO7A, green) and supporting cells (SOX2, red).

Scale bars = 20 μm.


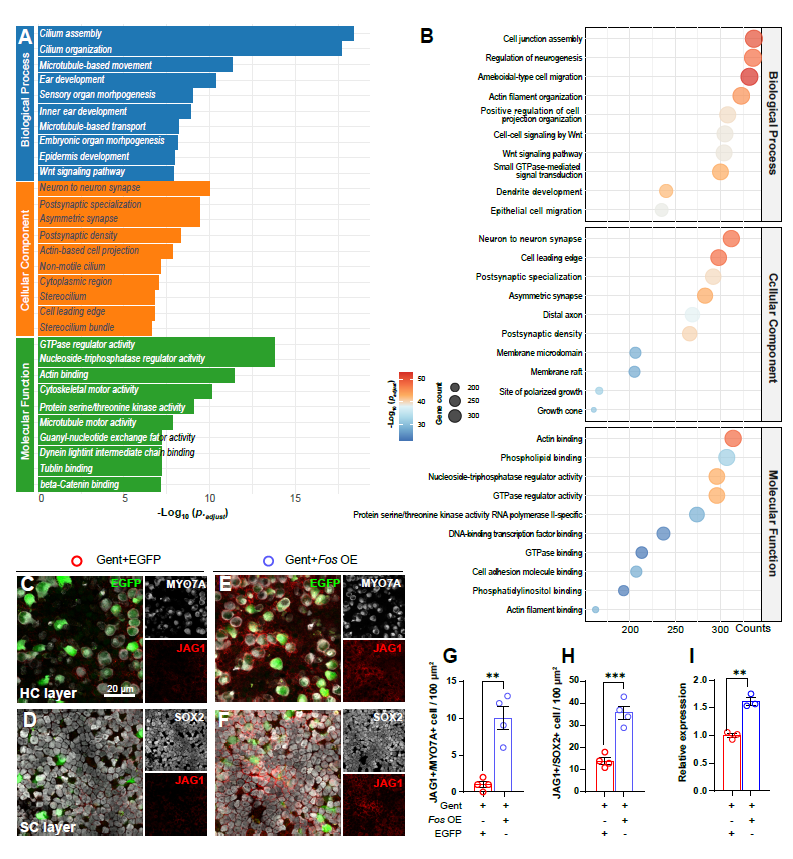


**Figure S4: Supplementary analysis of molecular pathways regulated by c-Fos.**

(A) Gene Ontology (GO) analysis of upregulated genes. Bar plot showing significantly enriched GO terms for genes upregulated in AAV-*Fos* OE-transduced utricles.

(B) GO analysis of c-Fos binding targets. Dot plot visualizing GO terms enriched for genes with c-Fos binding peaks identified by CUT&Tag analysis.

(C-H) *Fos* overexpression upregulates JAG1 protein expression in the utricular sensory epithelium. (C, D) Representative confocal images of utricles stained for JAG1 (white), MYO7A (red), and SOX2 (blue), from the (C-D) AAV-CMV-EGFP control group and (E-F) AAV-*Fos* OE group. For each condition, representative views of both the hair cell (HC) layer (C, E) and the supporting cell (SC) layer (D, F) are shown. (G-H) Quantification of (G) the number of JAG1+/MYO7A+ cells and (H) the number of JAG1+/SOX2+ cells, both showing a significant increase in the AAV-*Fos* OE group compared to the control (unpaired Student's t-tests; n = 4 per condition).

(I) qRT-PCR analysis confirms *Jag1* mRNA upregulation. Bar graph showing that *Jag1* mRNA levels are significantly elevated in AAV-*Fos* OE-transduced utricles compared to AAV-CMV-EGFP controls (unpaired Student's t-test; n = 4 per condition).

***p* < 0.01, ****p* < 0.001. Data are shown as the mean ± SEM.

Scale bars = 20 μm.


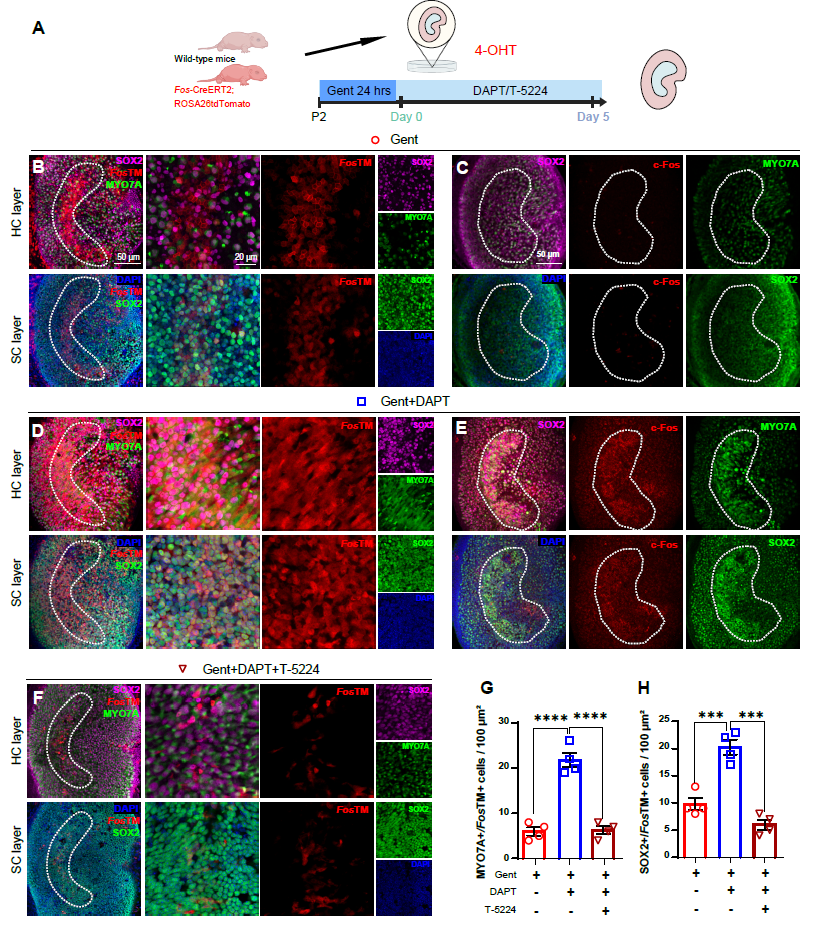


**Figure S5: Notch inhibition enhances both *Fos*-lineage labeling and c-Fos protein expression.**

(A) Schematic of the experimental design. P2 utricles from *Fos*TM mice were damaged with gentamicin. They were then cultured for 5 days in medium containing 4-OHT and EdU, under one of three conditions: gentamicin control, DAPT, or DAPT + T-5224.

(B-F) Representative confocal images comparing *Fos*-lineage labeling (*Fos*TM) and c-Fos protein immunofluorescence across the three treatment conditions. (B, D, F) display the *Fos*TM reporter (red), showing that DAPT treatment (D) markedly increases the number of labeled cells compared to the control (B), and this effect is reversed by co-treatment with T-5224 (F). Parallel immunofluorescence for c-Fos protein is shown in (C, E), confirming that DAPT treatment (E) also robustly induces c-Fos protein expression compared to the control (C).

(G-H) Quantification of *Fos*-lineage labeled cells. Bar graphs show that DAPT treatment significantly increased the number of (G) MYO7A+/*Fos*TM+ hair cells and (H) SOX2+/*Fos*TM+ supporting cells. This effect was reversed by co-treatment with the c-Fos inhibitor T-5224 (one-way ANOVA with Tukey’s HSD post-hoc test; n = 4 per condition).

****p* < 0.001, *****p* < 0.0001. Data shown as the mean ± SEM. Scale bars = 50 μm (low magnification) and 20 μm (high magnification).


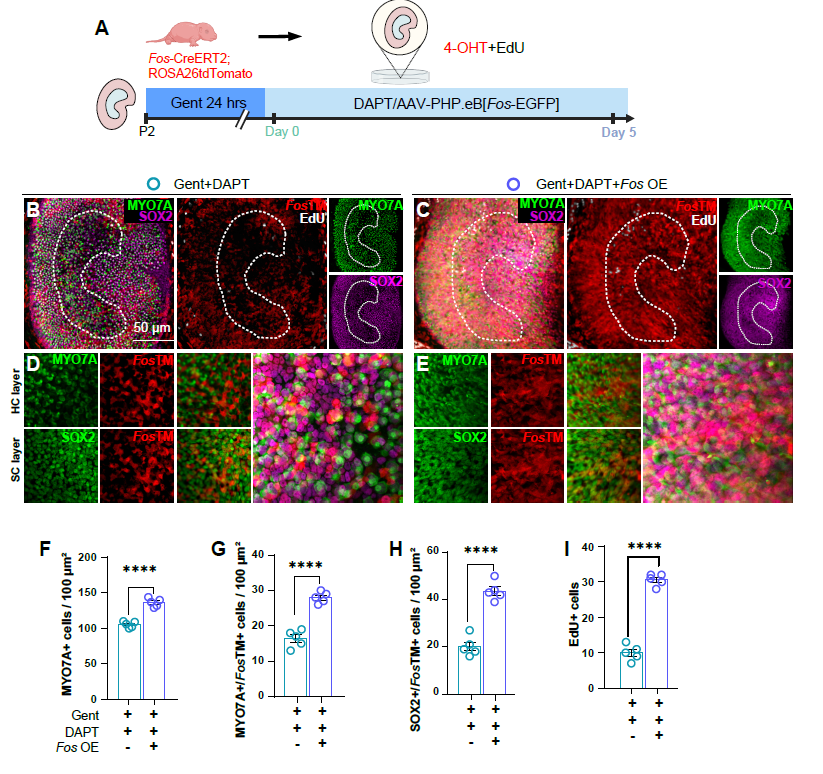


**Figure S6: Combined AAV-*Fos* OE and Notch inhibition synergistically enhances hair cell regeneration.**

(A) Schematic of the experimental design. P2 utricles from *Fos*TM mice were damaged with gentamicin. They were then cultured for 5 days in medium containing 4-OHT and EdU under different conditions: DAPT-alone, or a combination of DAPT + AAV-*Fos* OE.

(B-E) Representative confocal images of utricular sensory epithelia. Images show utricles from the (B) DAPT-alone group, and (C) the DAPT + AAV-*Fos* OE co-treatment group. (D-E) provides a higher magnification view of the striolar region, highlighting the increased number of MYO7A^+^/*Fos*TM^+^ cells.

(F-I) Quantification of (F) MYO7A^+^ cells, (G) MYO7A^+^/*Fos*TM^+^ cells, (H) SOX2^+^/*Fos*TM^+^ cells, and (I) EdU^+^ cells showed that all cell types increased after AAV-*Fos* OE/DAPT co-treatment compared to DAPT alone (unpaired Student's *t*-tests, n = 4 per condition).

*****p* < 0.0001. Data are shown as the mean ± SEM. Scale bars = 50 μm (low magnification) and 20 μm (high magnification).


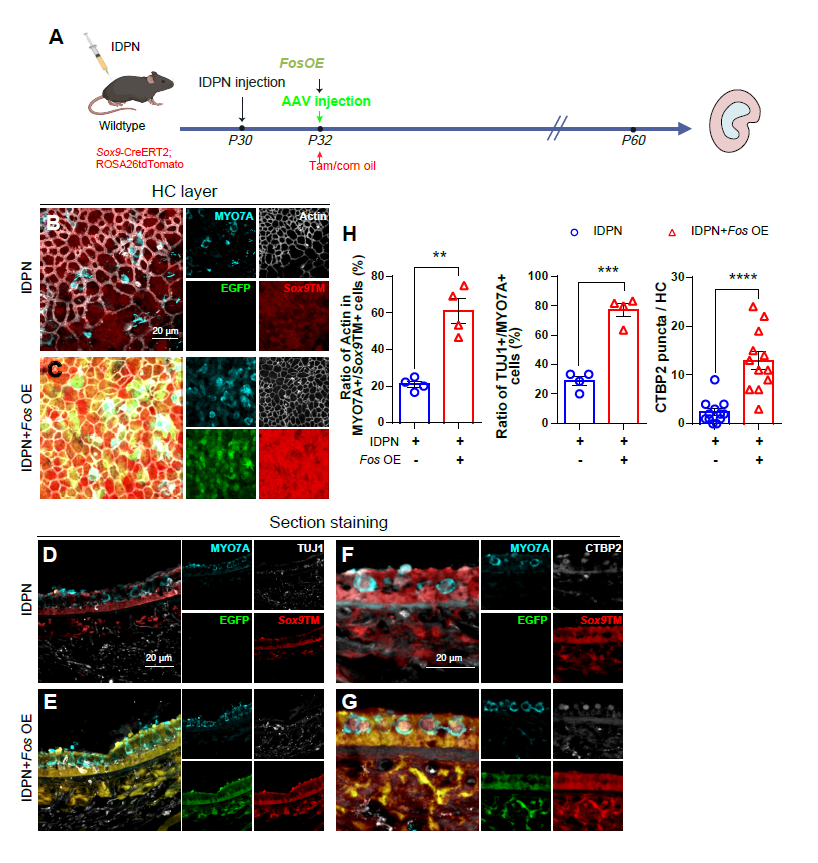


**Figure S7: *Fos* overexpression promotes the structural maturation and reinnervation of regenerated hair cells in vivo*.***

(A) Schematic diagram of the AAV-*Fos in vivo* intervention. Vestibular damage was induced in adult (*Sox9*TM) mice at P30, followed by AAV delivery at P32. Tissues were collected at P60 for analysis.

(B-D, b-d) *Fos*-mediated stereociliary bundle morphogenesis, neurite reinnervation patterns, and synaptic remodeling in regenerated hair cells as visualized by Actin, TUJ1, and CTBP2 co-labeled with MYO7A/EGFP/*Sox9*TM.

(E) Quantification of maturation and innervation metrics. Bar graphs show that, compared to the control group, *Fos* overexpression significantly increased the percentage of regenerated hair cells with actin bundles, the percentage of cells contacted by TUJ1^+^ neurites, and the density of CTBP2 synaptic puncta per hair cell (unpaired Student's t-tests, n = 4 per group).

***p* < 0.01, ****p* < 0.001, *****p* < 0.0001. Data are shown as the mean ± SEM. Scale bars = 20 μm.


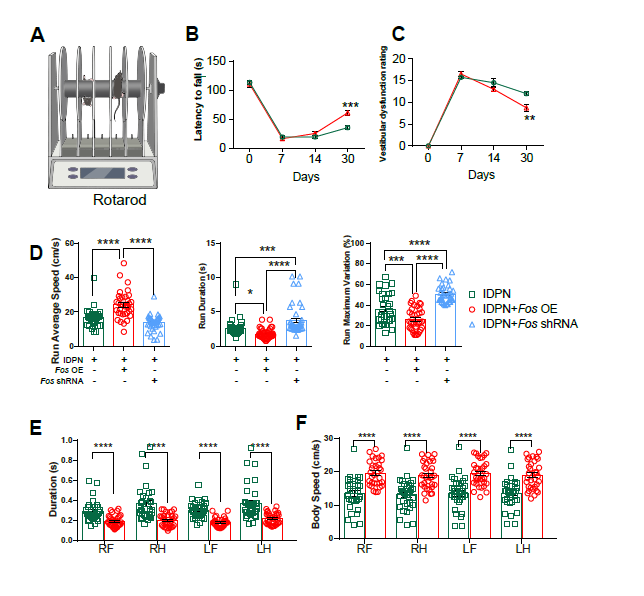


**Figure S8: *Fos* modulation affects motor coordination and gait performance after vestibular injury.**

(A) Schematic representation of the rotarod apparatus used to assess motor coordination and balance.

(B) Comparison of rotarod fall latency in the IDPN+*Fos* OE and IDPN+EGFP empty vector transfection groups at days 0, 7, 14, and 30 post-treatment. The IDPN+*Fos* OE group exhibited a significant improvement in rotarod performance over time compared to the IDPN+EGFP control group, particularly at days 14 and 30 (unpaired Student's *t*-tests, n = 4 per group).

(C) Vestibular Dysfunction Rating (VDR) scores, which assess vestibular dysfunction severity, in the IDPN+EGFP and IDPN+*Fos* OE groups at days 0, 7, 14, and 30. The IDPN+*Fos* OE group showed a faster reduction in VDR scores over time compared to the IDPN+EGFP group, indicating accelerated recovery (unpaired Student’s *t*-tests, n = 4 per group).

(D) Quantitative comparison of gait analysis parameters including run average speed, run duration, and run maximum variation (one-way ANOVA with Tukey’s HSD post-hoc test). The IDPN+*Fos* OE group exhibited better gait performance (higher speed, shorter run duration, and lower variation) compared to the IDPN+EGFP group, with AAV-*Fos* shRNA partially reversing these effects.

(E-F) Analysis of limb-specific movement speed and single-limb stance duration during locomotion for the right front (RF), right hind (RH), left front (LF), and left hind (LH) limbs (unpaired Student's *t*-tests). The IDPN+*Fos* OE group had reduced stance durations (E) and increased body speeds (F) across all limbs compared to the IDPN+EGFP group, indicating improved locomotion.

**p* < 0.05, ***p* < 0.01, ****p* < 0.001, *****p* < 0.0001. Data are shown as the mean ± SEM.
