# Supplementary figures and images for: Ototoxicity-induced c-Fos activation underlies the regenerative capacity of the vestibular sensory epithelia
Source: Cell Commun Signal. 2025 Oct 8;23:421. doi: 10.1186/s12964-025-02446-y (PMC12506291; doi:10.1186/s12964-025-02446-y)

## Slide 1
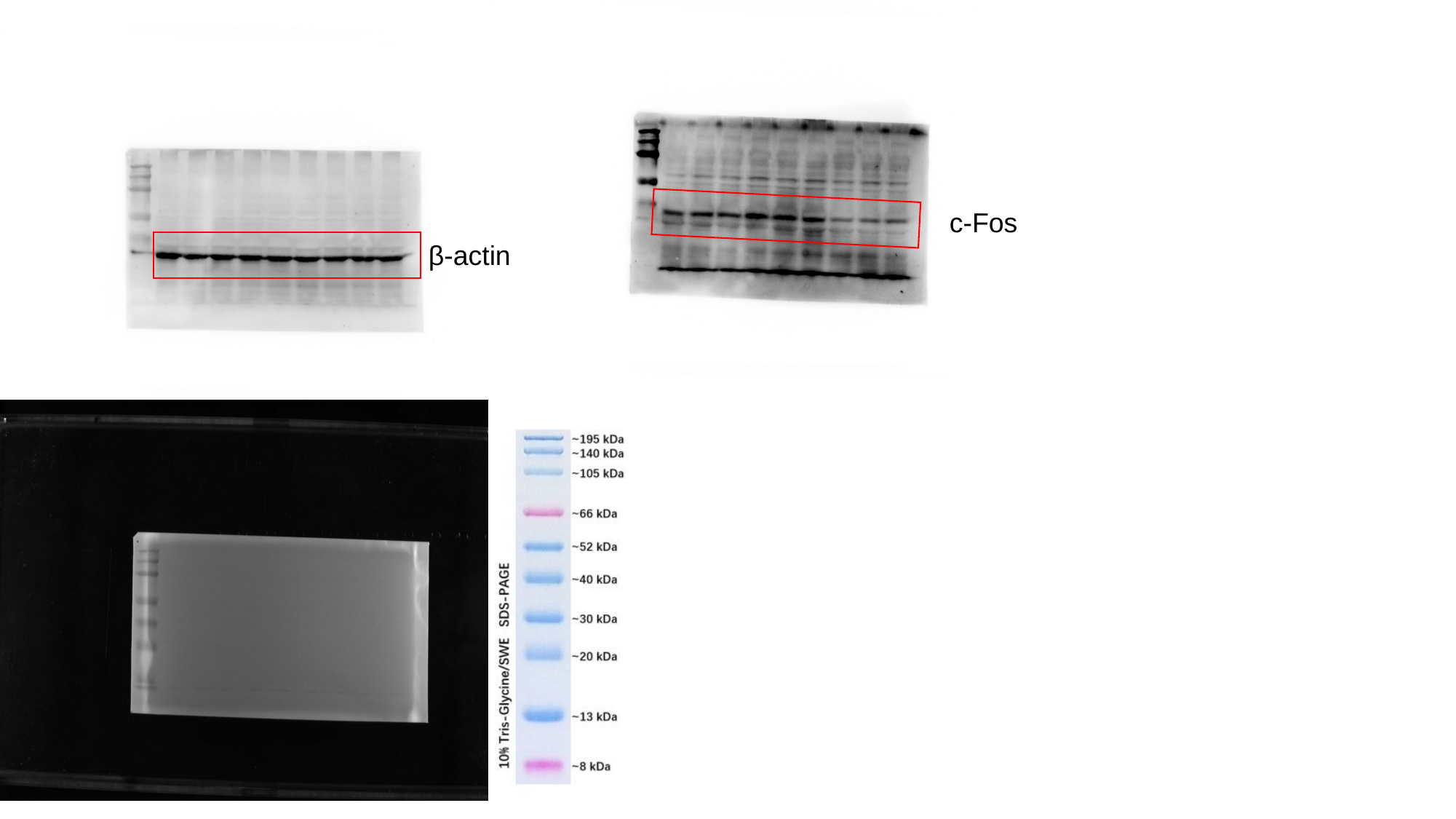

c-Fos
β-actin

Supplement: Supplementary file 2 — Supplementary Material 2. [file 12964_2025_2446_MOESM2_ESM.pptx]
